# Gate‐Tunable Remnant Anomalous Hall Effect in Dual‐‍Proximity Graphene Heterostructures

**DOI:** 10.1002/smsc.70404

**Published:** 2026-09-18

**Authors:** Sayooj Satheesh, Alexandre Bernard, Thomas Naimer, Ernst Knöckl, Takashi Taniguchi, Kenji Watanabe, Jaroslav Fabian, Alexander Holleitner, Christoph Kastl, Marko Burghard

**Affiliations:** ^1^ Max Planck Institute for Solid State Research Stuttgart Germany; ^2^ Walter Schottky Institute and Physics Department Technical University of Munich Garching Germany; ^3^ Munich Center for Quantum Science and Technology (MCQST) München Germany; ^4^ Institute for Theoretical Physics University of Regensburg Regensburg Germany; ^5^ Halle‐Berlin‐Regensburg Cluster of Excellence CCE University of Regensburg Regensburg Germany; ^6^ National Institute for Materials Science Tsukuba Japan

**Keywords:** coercivity, condensed matter physics, Curie temperature, ferromagnetism, graphene, Hall effect, heterojunction, physics

## Abstract

Monolayer graphene offers long spin coherence but lacks the intrinsic spin–orbit coupling (SOC) and magnetic exchange required to access anomalous Hall and topological transport. Simultaneously proximitizing graphene with a ferromagnet and a spin–orbit‐coupled semiconductor can, in principle, unlock responses inaccessible to either single‐proximity system, but an experimental demonstration has been missing. Here, we report a gate‐tunable, remnant anomalous Hall effect (AHE) in dual‐proximity Cr_2_Ge_2_Te_6_/graphene/WSe_2_ van der Waals heterostructures. Near charge neutrality, the trilayer exhibits a hysteretic transverse resistance with a remnant offset of up to Δ*R*_*xy*_ ≈ 250 Ω and a characteristic transport reversal field of ~20 mT, corresponding to an anomalous Hall conductivity of order e^2^/h that changes sign multiple times within a narrow energy window. The hysteresis vanishes above *T* ≈ 50 K, tracking the Curie temperature of Cr_2_Ge_2_Te_6_, and is absent in Cr_2_Ge_2_Te_6_/graphene bilayer controls. Scaling analysis of *σ*_*x* *y*_^AHE^ versus *σ*_*x* *x*_ rules out extrinsic skew‐scattering and side‐jump mechanisms near the Dirac point, while low‐energy model calculations reproduce the observed sign structure through Berry‐curvature hot spots that emerge only when exchange and SOC act together. Our results establish dual‐proximity engineering as a route toward gate‐tunable topological phases in graphene.

## Graphical abstract

Combining magnetic exchange from CGT and spin–orbit coupling from WSe_2_ in graphene produces a gate‐tunable remnant anomalous Hall response absent in control devices. This approach enables Berry curvature engineering toward electrically tunable topological transport in van der Waals heterostructures.

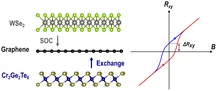

## 1 Introduction

Monolayer graphene exhibits exceptional spin and charge transport properties, with spin coherence lengths exceeding micrometers and charge mobilities exceeding 10 000 cm^2 ^V^−1 ^s^−1^ at room temperature [1]. Yet, at the same time, the lack of intrinsic spin–orbit coupling (SOC), which is as little as ~10 µeV [2], and of inherent magnetism preclude the realization of time reversal‐broken topological phases. Van der Waals (vdW) heterostructures offer a route to overcome both limitations by interfacing graphene with materials that impart strong SOC or magnetic exchange (ME) [3]. Of particular interest is the quantum anomalous Hall effect (QAHE), in which the interplay of SOC and ferromagnetic exchange opens a topological gap at the Dirac point and gives rise to dissipationless chiral edge transport without an external magnetic field [4].

Proximity‐induced SOC in graphene was first established through weak antilocalization (WAL) measurements on graphene/WS_2_ heterostructures, which revealed Rashba‐type SOC strengths of 1−10 meV [5]. Subsequent work demonstrated that this imprinted SOC enables spin‐valley locking, giant spin relaxation anisotropies (in‐plane/out‐of‐plane ratios of 10−70), gate‐tunable photogalvanic currents, and gate‐tunable spin‐to‐charge conversion via the spin Hall effect [6, 7, 8, 9, 10, 11], establishing that proximal SOC layers can be introduced without degrading the charge transport quality. In addition to the usual Rashba‐type SOC, the so‐called valley‐Zeeman, or Ising‐type SOC, is present in graphene transition metal dichalcogenide structures as well [5]. Their relative contributions can be tuned by the twist angle between graphene and the SOC layer, nowadays most commonly WSe_2_ [12], with vanishing valley‐Zeeman but persistent Rashba coupling for a specific twist angle of 30°.

In parallel, magnetic proximity has been explored by interfacing graphene with magnetic insulators, beginning with EuS [13] and extending to 2D vdW magnets such as CrI_3_ and CrBr_3_, where exchange splittings of up to ~10 meV have been reported [14, 15]. Recently, CGT/graphene heterostructures were shown to exhibit a robust field‐driven anomalous Hall effect (AHE) without sign reversal under gate tuning, confirming intrinsic magnetic proximity rather than stray‐field or multicarrier artifacts [16]. Towards the realization of topological phases, a very recent work reported evidence for a quantum spin Hall effect (QSHE) in graphene proximitized by the interlayer antiferromagnet CrPS_4_, including signatures of helical edge transport at zero field and a large nonquantized AHE under an applied field that vanishes above the Néel temperature (~38 K) [17]. However, all these single‐proximity systems require an applied magnetic field for AHE manifestation and lack spontaneous remnant hysteresis.

While these parallel advances have matured the study of individual proximity effects in graphene, a significant knowledge gap remains: the controlled combination of strong SOC and ferromagnetic exchange within the same graphene transport channel remains largely unexplored, both at zero external field as well as in the quantum Hall regime where the Landau level (LL) spectrum provides direct access to the band topology. Theory predicts that simultaneous SOC and exchange can drive phenomena inaccessible to single‐proximity systems, including giant Berry curvature near avoided crossings, Chern‐insulating (QAHE) phases, and electrically tunable topological transitions enabled by layer‐selective (“exsotic”) control of the proximity parameters [4].

Here, we report a switchable, remnant hysteretic transverse response in CGT/graphene/WSe_2_ vdW heterotrilayers up to nearly one order of magnitude larger than in our CGT/graphene bilayer control and previous studies on bilayers [16, 18]. We find that close to charge neutrality, the remnant Hall component is consistent with an intrinsic AHE from Berry curvature hotspots in the low‐energy band structure of the proximitized graphene that requires the simultaneous presence of exchange (imparted by CGT) and SOC (imparted by WSe_2_). Our results establish dual‐proximity engineering as a route to gate‐tunable anomalous Hall transport in graphene‐based vdW heterostructures [4].

**FIGURE 1:**
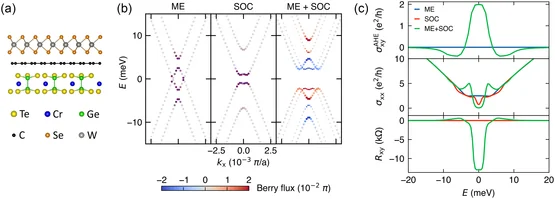
Dual proximity in Cr_2_Ge_2_Te_6_/graphene/WSe_2_ heterotrilayers. (a) In the heterostructure, Cr_2_Ge_2_Te_6_ (CGT) induces magnetic proximity exchange, and WSe_2_ induces SOC in the graphene layer. (b) Calculated low‐energy bands near the K (large markers) and K′ (small markers) valleys for three interaction scenarios: ME only (*λ*_E_ = −3.6 meV), SOC only (*λ*_R_ = 3.0 meV, *λ*_VZ_ = 2.0 meV), and combined exchange + SOC. A sublattice mass term *λ*_M_ = 0.9 meV is included in all cases. Marker colors encode the calculated Berry flux. (c) Intrinsic anomalous Hall conductivity *σ* *_xy_* ^AHE^, longitudinal conductivity *σ* *_xx_*, and Hall resistance *R* *_xy_* for the three scenarios in panel b at a carrier temperature of 4.2 K.

In the dual‐proximity heterostructure (Figure 1a), WSe_2_ serves to induce valley‐Zeeman‐type SOC in graphene [12, 19, 20, 21], while CGT provides magnetic proximity as an insulating ferromagnet with a *T*_C_ ≈ 65 K and sizable out‐of‐plane magnetic anisotropy [22, 23]. Structurally, CGT’s lattice constant (6.83 Å) is nearly commensurate with a √7‐expanded graphene supercell (√7 × *a*_g_ ≈ 6.51 Å), creating a low‐strain interface with only a 4.9% mismatch that promotes strong coupling [23, 24]. Moreover, its insulating character isolates the graphene transport response from parallel conduction pathways [25]. Theoretical studies predict that proximity of CGT induces sizable ME splitting in graphene’s band structure but very little SOC [24, 26]. Thereby, the dual proximitized trilayer architectures create a regime where individually controlled SOC and ME coexist and compete.

Figure 1b illustrates the effect of proximity‐induced ME, SOC, and their combination on graphene’s low‐energy Dirac spectrum, computed at both K and K′ valleys using an effective Hamiltonian with realistic proximity parameters (*λ*_R_ = 3.0, *λ*_VZ_ = 2.0, *λ*_E_ = −3.6, *λ*_M_ = 0.9 meV; see Section S8). Figure 1c translates these band structures into transport observables by computing the intrinsic anomalous Hall conductivity *σ* *_xy_* ^AHE^(*E*_F_), the longitudinal conductivity *σ* *_xx_* (*E*_F_), and the resulting Hall resistance *R* *_xy_* (*E*_F_) via tensor inversion for the same three scenarios. In the ME‐only limit, exchange lifts spin degeneracy but preserves valley‐contrasting Berry flux, so the valley‐summed AHE vanishes. In the SOC‐only limit, time‐reversal symmetry enforces exact cancelation between Kramers partners, again giving *σ* *_xy_* ^AHE^ = 0. Only in the combined ME + SOC case does exchange tilt the spin–orbit‐coupled states and lift the valley/Kramers compensation, producing Berry curvature hot spots near the Fermi level, a sizable intrinsic AHE of order kΩ, and, when *E*_F_ lies within the opened gap, a quantized plateau at 2e^2^/h characteristic of the QAHE.

## 2 Results

**FIGURE 2:**
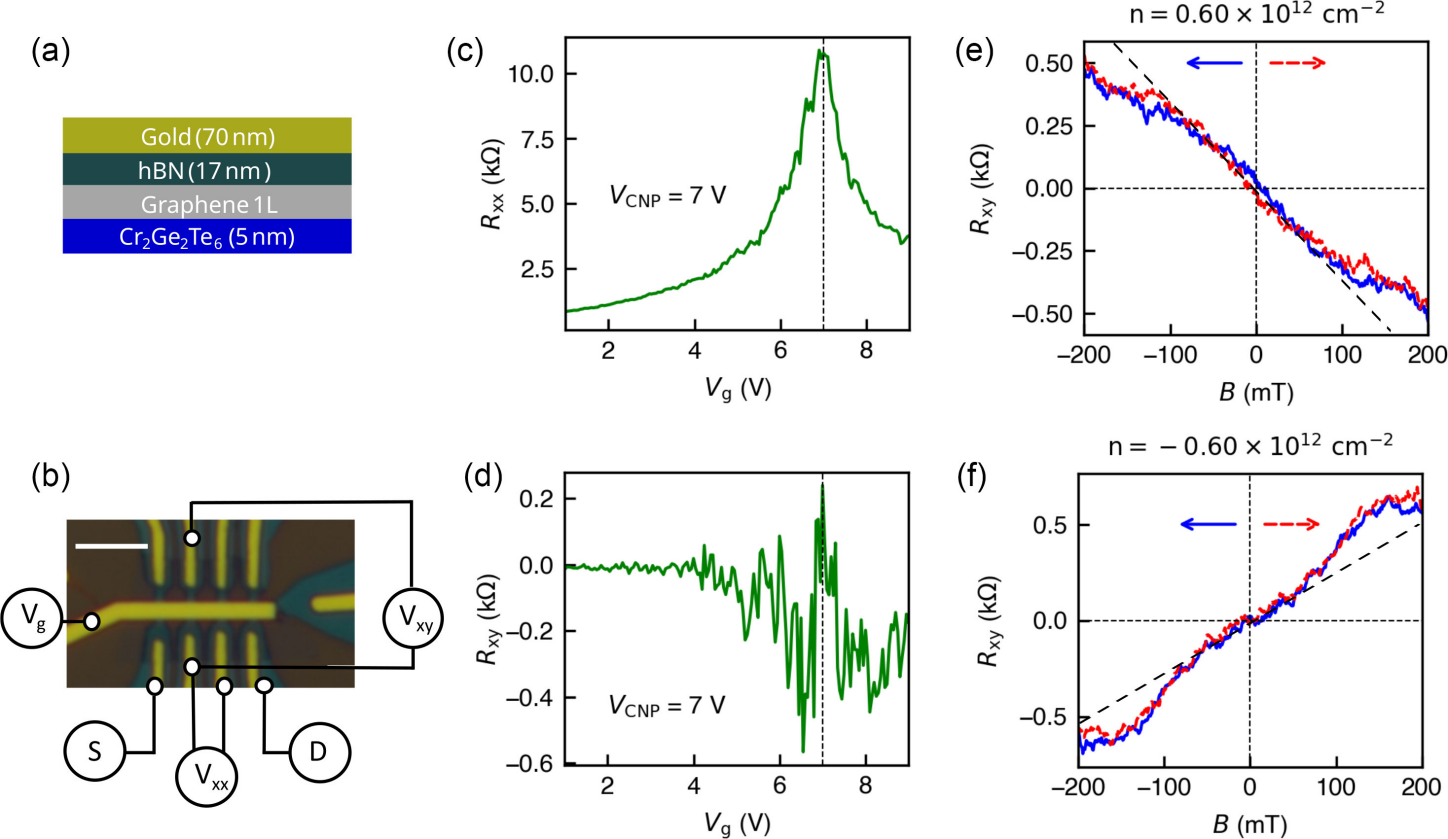
Cr_2_Ge_2_Te_6_/graphene heterostructure. (a) Schematic of the CGT/monolayer graphene heterostructure with hBN encapsulation and gold top gate. (b) Optical micrograph of the fabricated Hall‐bar device with source (S), drain (D), voltage probes (*V* *_xx_*, *V* *_xy_*), and top‐gate *V*_g_ connections indicated. Scale bar: 5 µm. (c) Gate‐voltage dependence of longitudinal resistance *R* *_xx_* at *B* = 0 T and *T* = 4.2 K. The charge neutrality point (*V*_CNP_ = 7 V) is indicated by the vertical dashed line. (d) Gate‐voltage dependence of transverse resistance *R* *_xy_* at *B* = 0 T and *T* = 4.2 K. (e,f) Transverse resistance *R* *_xy_* (*B*) as a function of out‐of‐plane magnetic field at *V*_g_ = 8.0 V and at *V*_g_ = 6.0 V, respectively. The corresponding carrier concentrations are indicated. The dashed red line is the mirror image of the solid blue line as obtained from the antisymmetrization procedure described in the main text.

We first characterize CGT/graphene bilayer samples, which serve as the control for isolating the role of WSe_2_‐induced SOC in the trilayer architecture. The layer sequence and an optical micrograph of the Hall bar device are shown in Figure 2a,b, respectively. The ferromagnetic transition of the CGT source crystal at *T*_C_ ≈ 65 K was confirmed by SQUID magnetometry [22, 23]. The transfer curve *R* *_xx_* (*V*_g_) shown in Figure 2c exhibits a single, well‐defined resistance maximum at the charge neutrality point (*V*_CNP_ ≈ 7 V, *T* = 4.2 K), indicating p‐type doping of the graphene channel at *V*_g_ = 0 V, potentially due to an interfacial charge transfer from graphene to CGT. From the gate capacitance of the hBN dielectric (*d* ≈ 17 nm), we estimate a residual hole density *n*_0_ ≈ 3.2 x 10^12^ cm^−2^ at *V*_g_ = 0 V and a corresponding hole mobility *μ* ≈ 2730 cm^2^/Vs (see Section S5). The sign and magnitude of the Dirac point shift are consistent with previous reports on graphene in proximity to CGT [16, 18].

Figure 2d shows the transverse resistance *R* *_xy_* recorded simultaneously at *B* = 0 T and *T* = 4.2 K. The data exhibit a finite signal over a broad gate range with pronounced oscillations near the CNP, superimposed on a slowly varying background. Since these *B* = 0 T contributions, most likely arising from probe misalignment, gate‐dependent current‐path inhomogeneity, or strain‐induced transverse voltages, are time‐reversal‐symmetric, they can be removed by field antisymmetrization, and we identify genuinely magnetization‐linked contributions exclusively from field‐hysteresis measurements. Accordingly, *R* *_xy_* (*B*) and *R* *_xx_* (*B*) are always presented as the antisymmetrized and symmetrized combinations of up‐ and down‐field sweeps, respectively; the complementary sweep direction is shown in dashed lines. Figure 2e,f display *R* *_xy_* (*B*) of the bilayer at *V*_g_ = 6.0 V and *V*_g_ = 8.0 V, corresponding to a substantial hole and electron doping of approximately 6 × 10^11^ cm^−2^,respectively. In this regime of single‐carrier transport, we find a reproducible nonlinearity of the Hall resistance, which we attribute to an AHE effect induced by the thin‐film magnetization of the nearby CGT layer. Consistently, the deviation from linear behavior (starting at 50 mT, see the dashed line in Figure 2e,f) appears at magnetic fields close to or below the saturation field of bulk CGT (within the range of 100−200 mT). Importantly, *R* *_xy_* (*B*) shows no clear remnant hysteretic component at *B* = 0 T within our experimental resolution, and this absence persists over the entire gate range *V*_g_ = 5–10 V, bracketing the bilayer CNP (*V*_CNP_ ≈ 7 V; Figure S2), similar to the earlier reports on bare CGT/graphene heterostructures [16].

**FIGURE 3:**
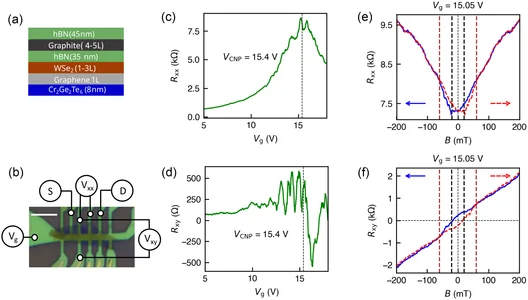
WSe_2_/graphene/CGT heterostructure. (a) Schematic of the CGT/graphene/WSe_2_ heterostructure with hBN encapsulation and graphite top gate. (b) Optical micrograph of the fabricated Hall‐bar device with source (S), drain (D), voltage probes (*V* *_xx_*, *V*_*x*y_), and top‐gate *V*_g_ connections indicated. Scale bar: 5 µm. (c) Gate‐voltage dependence of longitudinal resistance *R* *_xx_* at *B* = 0 T and *T* = 4.2 K. The charge neutrality point (*V*_CNP_ = 15.4 V) is indicated by the vertical dashed line. (d) Gate voltage dependence of transverse resistance *R* *_xy_* at *B* = 0 T and *T* = 4.2 K. (e) Symmetrized longitudinal resistance *R* *_xx_* (*B*), with the dashed vertical black lines indicating the characteristic reversal field at 20 mT and the dashed vertical red lines indicating the hysteresis‐closing field at 60 mT and (f) antisymmetrized transverse resistance *R* *_xy_* (*B*) near the CNP (*V*_g_ = 15.05 V) and *T* = 4.2 K. A pronounced low‐field hysteresis loop with remanent offset Δ*R* *_xy_* is visible.

Having established the bilayer baseline, we now turn to the WSe_2_/graphene/CGT trilayer (Figure 3a,b). To ensure comparable magnetic properties, we used a comparable CGT thickness in the trilayer sample (8 nm) and the bilayer sample (5 nm). The transfer curve *R* *_xx_* (*V*_g_) (Figure 3c) exhibits a resistance maximum at the CNP (*V*_CNP_ = 15.4 V, *T* = 4.2 K), indicating p‐type doping at *V*_*g*_ = 0 V similar to the bilayer device. A quantitative estimate of the hole density and mobility at *V*_*g*_ = 0 V, obtained from a two‐carrier fit, yields *n*_0_ = 3.2 × 10^12^ cm^−2^ and 6000 cm^2^/Vs, in good agreement with independent values extracted from the Shubnikov‐de Haas (SdH) oscillation period (*n*_0_ = 3.41 × 10^12^ cm^−2^, Figure S5). We note that this low‐temperature mobility is similar to WSe_2_/graphene/SiO_2_ [27] and hBN/graphene/CrPS_4_ [17] devices. As WSe_2_ is generally known to be a high‐quality encapsulation layer [19], we tentatively conclude that the magnetic proximity layer tends to introduce a disorder landscape that is similar to Si/SiO_2_ used in many “first‐generation” graphene devices [28].

Figure 3d shows *R* *_xy_* (*V*_g_) of the trilayer at *B* = 0 T and *T* = 4.2 K. As in the bilayer (Figure 2d), a finite transverse response is present over the full gate range, with sign reversal near *V*_CNP_ ≈ 15.4 V and pronounced oscillations superimposed near the CNP. For the same reasons discussed there, i.e., probe misalignment, gate‐dependent current paths, and strain‐induced time reversal‐symmetric transverse voltages, this *B* = 0 T background does not by itself conclusively indicate a magnetization‐linked AHE, and we focus in the following on field sweeps, which we antisymmetrize and symmetrize for *R* *_xy_* (*B*) and *R* *_xx_* (*B*), respectively.

At *V*_g_ = 15.05 V (slightly hole‐doped relative to *V*_CNP_) and *T* = 4.2 K, the symmetrized longitudinal resistance *R* *_xx_* (*B*) of the trilayer exhibits a clearly resolved butterfly‐like hysteresis (Figure 3e). Shallow resistance minima appear near ±20 mT, which coincides with a characteristic low‐field reversal scale of ~20 mT (black dashed lines in Figure 3f), and the up‐ and down‐sweep traces merge for |*B*| ≳ 60 mT, which matches the merging of the corresponding *R* *_xy_* traces (red dashed lines in Figure 3f). At higher fields, R_xx_ increases monotonically, reaching ~30% above the low‐field minima at |*B*| = 200 mT, consistent with the positive two‐carrier magnetoresistance expected near charge neutrality [29]. Similar features with modest gate‐dependent variations are observed at nearby voltages (Figure S3). The butterfly‐like *R* *_xx_* (*B*) response, the shallow minima near ∣*B*∣ ≈ 20 mT, and the merging of the sweep directions near ∣*B*∣ ≈ 60 mT indicate that the longitudinal transport is correlated with the magnetic reversal and domain evolution of the CGT flake. Because *R* *_xx_* is a dissipative response, its microscopic origin cannot be determined from these data alone. Possible contributions include domain wall‐related scattering [30, 31], spatial variation of the proximity exchange field, and stray fields from CGT domains that modify the local magnetoresistance. In addition, because the measured resistivities are related through the inversion of the conductivity tensor, a field‐dependent transverse conductivity can contribute to the observed variation of *R* *_xx_*. Regarding the possible influence of stray fields, it should be noted that previous work argued against a dominant stray field origin on the basis of a control experiment in which a ~5 nm hBN spacer inserted under ambient conditions between CGT and graphene suppressed the AHE [16]. However, CGT is highly air‐sensitive and develops oxide and nonmagnetic layers of comparable thickness upon ambient exposure [32], which complicates the interpretation of the spacer control. We therefore treat both stray field‐coupling and domain wall‐related scattering as plausible magnetization‐linked background mechanisms.

In marked contrast to the bilayer, where *R* *_xy_* (*B*) shows no resolvable remnant component (cf. Figure 2e,f), the trilayer’s transverse response near the CNP (Figure 3f) exhibits a pronounced low‐field hysteresis loop of several hundred ohms centerd around *B* = 0 T. The field‐sweep displays a finite, reproducible offset Δ*R* *_xy_* at *B* = 0 T. At higher |*B*|, *R* *_xy_* becomes approximately linear, consistent with the ordinary Hall background as in the bilayer case. We note that three additional independently fabricated trilayer devices exhibit qualitatively identical low‐field hysteresis loops with Δ*R* *_xy_* in the range of 100–200 Ω near charge neutrality (see Table S1 and Figures S7 and S12), confirming that the remnant component is a reproducible property of the CGT/graphene/WSe_2_ trilayer architecture.

**FIGURE 4:**
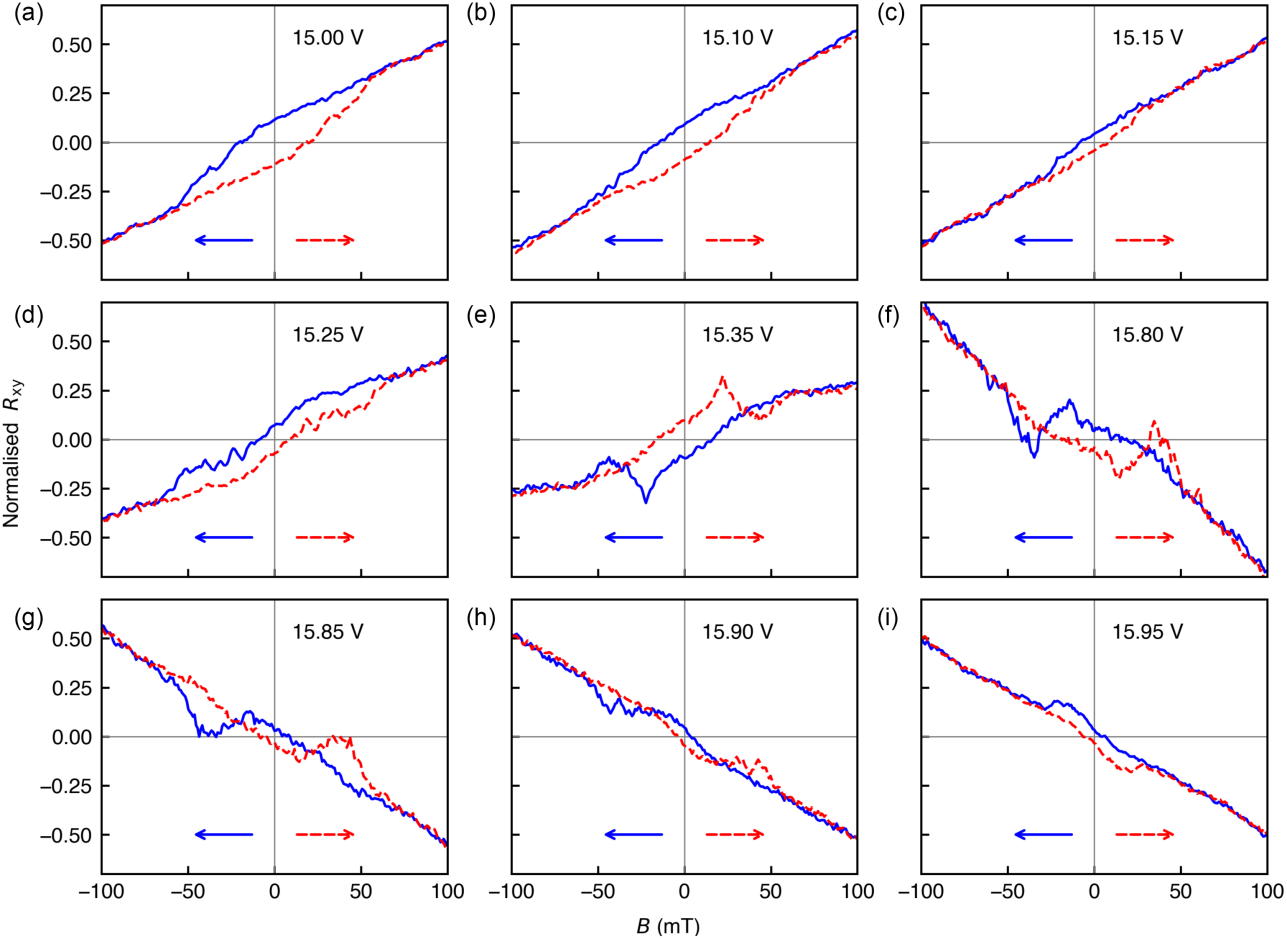
Gate‐dependent evolution of the hysteresis loop for the trilayer. (a–i) Normalized Hall resistance in the magnetic field range from −100 to 100 mT at the indicated gate voltages and *T* = 4.2 K.

In Figure 4, we compile normalized Hall resistance traces covering a narrow gate window *V*_g_ = 15.00–15.95 V around *V*_CNP_ = 15.4 V. We emphasize four notable features (see Figure S4 for data over a wider gate voltage range). First, the sign of the approximately linear high‐field background reverses upon crossing the CNP (e.g., comparing *V*_g_ = 15.40 and 15.75 V), consistent with the ordinary Hall response changing sign with the carrier type. Second, the low‐field hysteretic contribution is strongly *V*_g_‐dependent and deviates from a simple square loop, as manifested by additional overshoot features at selected gate voltages (e.g., *V*_g_ = 15.35, 15.75, and 15.80 V). These are possibly due to either the superposition of AHE contributions from regions with different local coercivities, gate‐dependent domain reversal sequence, or the complex magnetization patterns deviating from spatially uniform magnetization, such as labyrinth and stripe domains observed in CGT crystals of comparable thickness [33]. Third, at certain voltages (e.g., near *V*_g_ = 15.8 V), the branch‐crossing field is smaller than the field at which the sweep‐dependent contribution becomes unresolved and *R* *_xy_* becomes approximately linear. This separation is consistent with multistep reversal and/or a distribution of local magnetic switching fields. Finally, and most strikingly, the sign of the magnetization‐correlated deviation from the ordinary Hall background can invert with gate‐voltage changes as small as 50 mV (e.g., between *V*_g_ = 15.25 and 15.30 V), which would be consistent with a rapid evolution of Berry curvature through the Fermi energy near charge neutrality, as anticipated from the calculated *σ* *_xy_* ^AHE^(*E*_F_) (Figure 1c).

Accordingly, the sensitivity of Δ*R*_xy_ and the loop shape to the gate voltage within a narrow window around the CNP may reflect two entangled effects. On the electronic side, the gate tunes E_F_ through the Berry curvature landscape of the proximitized graphene bands (Figure 1c), where even a 50‐mV shift can move the Fermi level between regions of opposite *σ* *_xy_* ^AHE^. On the magnetic side, interfacial charge redistribution at the CGT/graphene interface may modify domain nucleation thresholds and the transport‐derived switching‐field distribution in the 8 nm CGT flake. Ionic gating studies at much higher doping (~10^14^ cm^−2^) have shown that charge doping can tune both the magnetization loop and the magnetic anisotropy of few‐layer CGT [34, 35], and DFT calculations indicate that even moderate carrier injection enhances the exchange interaction in monolayer CGT [36]. Although our solid‐state gate induces substantially lower carrier densities in graphene (~10^12^ cm^−2^), the analysis of the carrier density‐gate voltage relation in Section S6 reveals substantial charge trapping at the CGT/graphene interface. Since a fraction of the gate‐induced charge resides at this interface rather than in the graphene channel, its local electrostatic influence on the CGT layer may be nonnegligible, and even a partial tuning of the domain configuration in the few‐nm flake could shift local coercivities and contribute to the observed loop fine structure.

**FIGURE 5:**
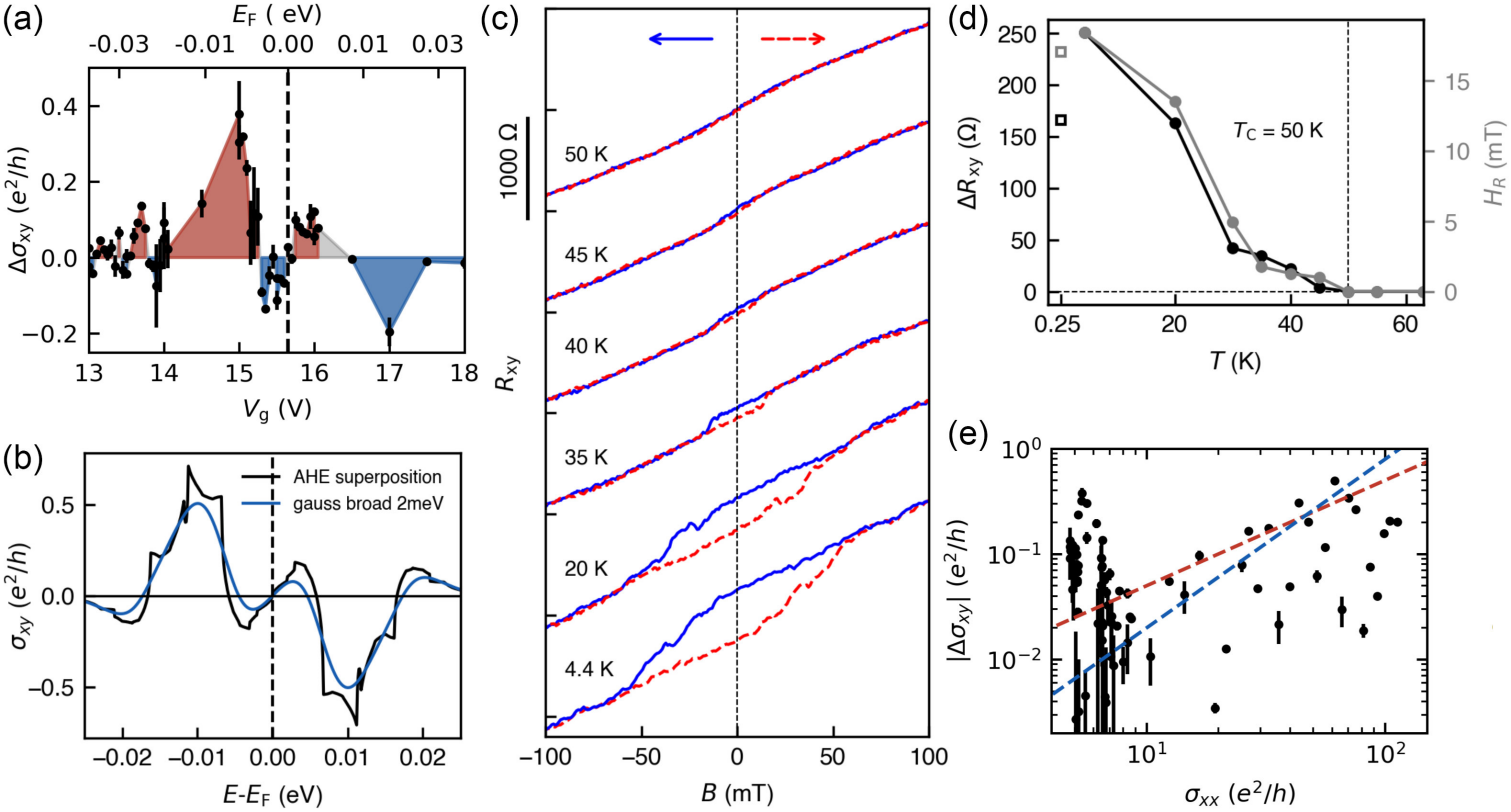
Gate‐ and temperature‐dependence of the Hall effect hysteresis with dual proximity. (a) Gate dependence of the anomalous Hall conductivity at *B* = 0 T quantifying the remnant contribution to *σ* *_xy_*. The top axis denotes the Fermi level calculated from the measured density. (b) Theoretical calculation of the intrinsic contribution to *σ* *_xy_* for a disordered, proximitized graphene channel. (c) Temperature evolution of *R* *_xy_* (*B*) at *V*_g_ = 15.0 V. (d) Temperature dependence of ∣Δ*R* *_xy_* ∣ and characteristic transport reversal field H_R_ extracted from c, showing a progressive suppression and disappearance of the hysteretic component around *T* ≈ 50 K. The squares denote measurements at 250 mK from a separate cooling cycle. (e)  Scaling of Δ*σ* *_xy_* versus *σ* *_xx_*. The dashed lines are guides to the eye for linear scaling *σ* *_xx_* ^1^ (red, clean limit of extrinsic contribution) and superlinear scaling *σ* *_xx_* ^1.6^ (blue, dirty limit). The data points close to the charge neutrality point (small *σ* *_xx_*) deviate from both scaling laws.

Figure 5a summarizes the gate dependence of the remnant anomalous Hall conductivity, i.e., its zero‐field hysteresis, calculated via $\Delta \sigma_{xy}=\Delta \rho_{xy}/({\rho_{xx}}^{2}+{\left(\Delta \rho_{xy}\right)}^{2})$, whereby $\Delta \rho_{xy}$ denotes the remnant AHE as introduced above and ${\rho_{xx}}^{2}$ denotes the regular longitudinal resistivity at *B* = 0 T. The anomalous Hall conductivity shows a value on the order of e^2^/h close to the Dirac point as well as multiple sign reversals within a narrow range of Fermi energies (about 20 meV). Note that we took care to calibrate *V*_g_ versus *E*_F_ close to the Dirac point, taking into account both a two‐carrier model and deviations from a constant gate capacitance due to interface trap states (Figure S5). A simple model of the intrinsic AHE contribution, as illustrated in Figure 1, would predict only two sign reversals across charge neutrality. However, we can qualitatively model our data by considering a situation where multiple domains with different strengths of the exchange parameters and different Fermi level alignments are present within the measured area of the sample. Given the complex behavior of magnetic domains in thin CGT layers [33] and the comparably moderate mobility (~6000 cm^2 ^V^−1 ^s^−1^), a disordered electronic and magnetic potential landscape seems plausible. The results of such a model calculation of the intrinsic, Berry curvature‐induced AHE for realistic exchange parameters are displayed in Figure 5b showing sharp resonances due to the Berry curvature hot spots. With a disorder broadening of 2–3 meV (as discussed below in the context of further quantum Hall measurements), this phenomenological model reproduces the qualitative magnitude and gate‐dependent sign structure of the measured anomalous Hall conductivity. It is not intended as a unique quantitative fit since the measured response likely reflects spatial variations in the local Fermi level, exchange coupling, and magnetic‐domain configuration. The temperature evolution of Δ*R* *_xy_* and *H*_R_ (Figure 5c,d) provides further evidence for an intrinsic origin linked to the magnetic proximity. The AHE appears sharply below 50 K, which is close to the measured bulk value of *T*_C_ ≈ 65 K and consistent with *T*_C_ ≈ 55‍–‍60 K reported for exfoliated CGT flakes of comparable thickness [37, 38]. With decreasing temperature, Δ*R* *_xy_* increases further in a linear fashion, which we attribute to the reduction of thermal smearing (1 meV exchange coupling corresponds to about 12 K thermal energy) and then starts to saturate below 4.2 K (squares in Figure 5d correspond to a measurement taken at 250 mK in a separate cooling cycle).

## 3 Discussion

Three complementary observations support the interpretation of the near‐CNP Hall response as predominantly intrinsic and Berry‐curvature driven. First, the scaling plot in Figure 5e provides the sharpest mechanistic discrimination. It depicts the remnant anomalous Hall conductivity versus longitudinal conductivity evaluated across the full gate range, with dashed lines marking the linear (*α* = 1) and superlinear (*α* = 1.6) trends expected for extrinsic skew scattering and the dirty metal (side‐jump‐dominated) regime, respectively [39, 40, 41]. Away from charge neutrality, where σ_xx_ is large, σ_xy_^AHE^ scales as *σ* *^α^* *_xx_* with *α* ≈ 1‍–‍1.6, bracketing these two extrinsic limits. Crucially, near the CNP, the data lie well above both extrapolated scaling lines. Within the standard metallic‐regime extrinsic scaling picture (*σ* *_xy_* ^AHE^ ∝ *σ* *_xx_* *^α^* with α ≥ 1), this is incompatible with a purely extrinsic origin. Specifically, for *α* > 1 (dirty/side‐jump limit), *σ* *_xy_* ^AHE^/*σ* *_xx_* → 0 as *σ* *_xx_* → 0, while for *α* = 1, the skew prefactor is of order (*λ*_SOC_/E_F_)^2^ ≪ 1 at our SOC scales, which is far too small to account for the observed *σ* *_xy_* ^AHE^ of order e^2^/h. The upward deviation, therefore, requires an intrinsic band‐geometry contribution decoupled from *σ* *_xx_*, which is the hallmark of Berry curvature‐dominated transport.

Second, the value *σ* *_xy_* ^AHE^ approaching e^2^/h near the Dirac point is difficult to reconcile with a purely extrinsic interpretation. In Rashba‐type 2D models, skew‐scattering and side‐jump contributions scale roughly as *σ* *_xy_* ^ext^ ∝ (*λ*_R_/*E*_F_)^2^ *σ*_xx_, implying strong suppression when *λ*_R_ ≪ *E*_F_ [39, 42]. Moreover, in 2D systems with both chiral subbands occupied, skew scattering can be strongly suppressed or vanish [43]. Given our inferred proximity SOC scale (~1–3 meV), extrinsic contributions are therefore expected to remain well below e^2^/h in the relevant density range. By contrast, intrinsic Berry curvature concentrated near meV‐scale avoided crossings can generate *σ* *_xy_* contributions of order e^2^/h, consistent with the Berry curvature hot spots in Figure 1b and with QAHE/AHE theories for exchange + SOC Dirac bands [4, 44]. The observed *σ* *_xy_* ^AHE^ ≈ e^2^/h is difficult to reconcile with the standard extrinsic mechanisms expected at the inferred SOC scales.

Third, the most direct topological fingerprint is the gate‐induced sign reversal of *σ* *_xy_* ^AHE^ (Figures 4 and 5a). The Hall response changes sign with gate voltage steps as small as Δ*V*_g_ ≈ 50 mV (Δ*E*_F_ ≈ 0.5 meV near the CNP) without an intentional reversal of the applied field history. In the simplest extrinsic pictures, the AHE sign is expected to track the magnetization direction, such that rapid gate‐driven sign inversions at a fixed M are not naturally anticipated [39]. Although sign changes can in principle arise from disorder‐ and scattering‐specific extrinsic mechanisms in 2D models [45], the observed multiple sign reversals within a narrow ~20 meV window are consistent with *E*_F_ traversing a structured Berry curvature landscape in exchange‐ and SOC‐split Dirac bands (Figure 1c), as reproduced by the intrinsic calculation in Figure 5b once disorder broadening (or equivalently thermal smearing) is included. Taken together, these three lines of evidence establish that the observed remnant AHE resides in a nonquantized QAHE precursor regime, in which dual proximity yields a large, gate‐tunable Berry curvature‐driven Hall response. The conditions required to advance to a fully quantized state are identified as follows.

The CGT/graphene bilayer serves as an important control. By sharing the same ferromagnetic proximity layer while lacking the WSe_2_ SOC source, the bilayer control shows that CGT‐related magnetism or exchange alone does not yield a resolvable remnant Hall response of comparable magnitude and gate dependence under our measurement conditions. The emergence of a finite Δ*R* *_xy_* peak (~250 Ω at *V*_g_ ≈ 15 V, *T* = 4.2 K) only in the trilayer isolates the one differing ingredient, namely, the strong interfacial SOC from WSe_2_, which enables Berry curvature‐driven *σ* *_xy_* ^AHE^ in the exchange‐split, spin–orbit‐coupled Dirac bands. Importantly, we verified that our samples are aligned with twist angles different from 30°, such that both Rashba‐ and valley Zeeman‐type couplings are present at a comparable scale [12, 46]. To this end, we determined the orientation of WSe_2_ from polarized second harmonic generation [47] and of the graphene layer by polarized Raman spectroscopy [11, 48].

To quantify the magnetostatic stray field contributions, we estimate an upper bound on the ordinary Hall offset they could generate near the CNP. Using *M*_s_ ≈ 50 emu cm^−3^ for CGT [23], the characteristic field scale is *μ*_0_ *M*_s_ ≈ 60 mT. Approximating the local ordinary Hall response by the measured slope of *R* *_xy_* (*B*) near the CNP, *R* *_H_* ^eff^(*V*_g_), yields an offset *R* *_H_* ^eff^ *μ*_0_ *M*_s_ of ≤~20 Ω. For a uniformly magnetized thin film, the laterally averaged out‐of‐plane stray field is expected to be much smaller over areas large compared to the film thickness (geometry‐dependent demagnetizing fields) [49]. Taken together, these bounds suggest that ordinary Hall stray‐field offsets are unlikely to account for the observed Δ*R*_xy_ ≈ 250 Ω near the CNP, although domain/edge‐fringing fields [50] could still contribute at a smaller level.

We note that the impact of film thickness and interfacial proximity on the magnetic phase diagram of CGT deserves careful consideration. First, to achieve a remnant hysteresis in CGT, the film thickness is critical. Bulk CGT is a soft perpendicular ferromagnet which displays a saturation field within the range of 100–200 mT with very little to no remnant magnetization at zero field. As the thickness is decreased (on the order of 50 nm), the interplay of different magnetic textures, including stripes, labyrinths, and bubbles, produces a magnetic history‐dependent phase diagram [51] and slanted or bow‐tie‐shaped hysteresis curves [52], still with very little to no remnant magnetization at zero field. For the thickness used in our study (on the order of 10 nm and below), multiple studies have reported remnant zero‐field magnetization and nearly square‐shaped hysteresis loops, indicative of a regime where the magnetization is driven by a few extended domains [33, 50, 53]. Second, proximity‐induced modifications of CGT magnetism have been reported in other heterostructures: in CGT/NiO, enhanced out‐of‐plane magnetic anisotropy and an increased critical temperature were observed [37]. A similar modification of the magnetic properties of CGT in our devices cannot be completely excluded a priori based on transport measurements alone. However, because CGT and WSe_2_ are separated by graphene, any such influence would have to be indirect, for example, through charge redistribution, strain, or graphene‐mediated interfacial coupling. Such effects may contribute to the gate‐dependent reversal‐field scale and fine structure of the hysteresis loops. However, they do not by themselves naturally explain the multiple reversals of *σ* *_xy_* ^AHE^ within a narrow energy range near charge neutrality. These reversals are instead consistent with the Fermi level traversing a structured Berry‐curvature landscape generated by the combined action of CGT‐induced exchange and WSe_2_‐induced SOC.

Finally, we address the factors that limit the advance from this QAHE precursor regime to a fully quantized state. From analysis of the high‐field LL sequence, a representative parameter set yields *λ*_R_ ≈ 3.0 meV, *λ*_VZ_ ≈ 2.0 meV, *λ*_E_ ≈ −3.6 meV, and *λ*_M_ ≈ 0.9 meV (see Section S9), consistent with the experimental range of 1‍–‍10 meV reported for graphene/WSe_2_ [19, 20, 21, 54]. Despite these sizable proximity scales, we find at most Δ*R*_xy_ ≈ 250 Ω below 4.2 K (~1% of h/e^2^), indicating appreciable dissipative bulk conduction that short‐circuits possible edge transport. Two factors compound to prevent quantization, specifically (i) disorder broadening *σ* ≈ 2–3 meV, comparable to the proximity scales themselves, allowing charge‐puddle percolation through the bulk, and (ii) spatial nonuniformity of the proximity couplings (e.g., twist or registry variations) produces position‐dependent gap sizes that impede the formation of a single robust chiral edge channel. Given the large charge transfer from CGT and the substantial interface trap density (*D*_it_ ≈ 2.9 × 10^13^ cm^−2^ eV^−1^, Section S6) observed in both bilayer and trilayer devices, the CGT/graphene interface, rather than the WSe_2_/graphene interface, is the dominant limiting factor.

## 4 Outlook

Our results open several concrete directions for future work. Achieving quantization requires disorder broadening well below 1 meV and improved electrostatic homogeneity near charge neutrality. These targets can be addressed through cleaner CGT exfoliation and optimized transfer protocols, guided by spatially resolved electrostatic mapping (e.g., Kelvin‐probe microscopy). Twist‐angle engineering offers a systematic route to tune the proximity exchange and SOC magnitudes, motivating angle‐resolved assembly correlated with LL spectroscopy and gate‐dependent transport [55]. Beyond quantization, integrating dual‐proximitized graphene with superconducting contacts could open a route to exchange‐ and SOC‐engineered Andreev physics and gate‐tunable topological superconducting phases [56].

## 5 Methods

### 5.1 Device Fabrication

Monolayer graphene, few‐layer WSe_2_ (purchased from HQ Graphene, Netherlands), and hBN (NIMS Japan) flakes were mechanically exfoliated using the standard Scotch tape method under ambient conditions onto SiO_2_ (300 nm)/Si substrates. Few‐layer CGT (HQgraphene) was exfoliated in an Ar‐filled glove box with O_2_ and H_2_O levels of 1 and 1.5 ppm, respectively, to prevent degradation. Monolayer graphene regions were identified using optical contrast. For the dry‐transfer process using a poly(bisphenol A carbonate) (PC)/polydimethylsiloxane (PDMS) stamp, first the top hBN (45 nm) was picked up, followed by graphite, middle hBN (35 nm), WSe_2_ (few layers), monolayer graphene, and few‐layer Cr_2_Ge_2_Te_6_ (<10 nm). The pick‐up steps were performed at 80–100 °C, and the heterostructure was released onto prepatterned marker substrates at 170 °C. Following release, the PC layer was dissolved in chloroform for 15 min, and the stack was subsequently cleaned in acetone and isopropanol. Device fabrication involved multiple stages of e‐beam lithography and reactive‐ion etching. First, one‐dimensional edge contacts were fabricated by etching the heterostructure, followed by deposition of a Cr/Au bilayer (5/80 nm). Finally, the device was etched into Hall bar geometry via reactive ion etching using a CHF_3_/O_2_ (40 sccm/4 sccm) gas mixture at *T* = 43 °C using 60 W RF power.

### 5.2 Electrical Transport Measurements

Electrical transport measurements were performed in a He‐3 cryostat (Oxford Instruments) equipped with a variable temperature insert (VTI), covering a temperature range from 250 mK to 300 K with applied magnetic fields up to 12 T. Longitudinal resistance (*R* *_xx_*) and Hall resistance (*R* *_xy_*) were measured using standard four‐probe techniques using Signal Recovery DSP7265 lock‐in amplifiers with low‐frequency excitation (7.77 Hz) and currents of 100 nA. The DC gate voltages were applied via a Keithley 2400 source meter.

### 5.3 Data Processing and Analysis

For each pair of forward and reverse field sweeps at each gate voltage *V*_g_, the antisymmetrized Hall signal is obtained as *R* *_xy_* (*B*) = ½ [*R* *_xy_* ^↑^(*B*)−*R* *_xy_* ^↓^(−*B*)], while the symmetrized longitudinal signal is *R* *_xx_* (*B*) = ½ [*R* *_xx_* ^↑^(*B*) + *R* *_xx_* ^↓^(−*B*)]. We plot *R* *_xx_* (*B*) and *R* *_xy_* (*B*) as solid lines. The twin curves *R* *_xx_* (−*B*) and −*R* *_xy_* (−*B*), which are the even and odd counterparts obtained from the complementary sweep combination ½[*R* *_xx_* ^↓^(*B*) + *R* *_xx_* ^↑^(−*B*)] and ½[*R* *_xy_* ^↓^(*B*)−*R* *_xy_* ^↑^(−*B*)], respectively, are plotted as dashed lines. Any splitting between the solid and dashed traces encodes the remnant hysteretic component of the response.

## Funding

This work was supported by Deutsche Forschungsgemeinschaft (Grants 443274823, 418933089, 522732992, 443416183, and 314695032), Munich Center for Quantum Science and Technology (Grant 2111‐390814868), European Union’s Horizon Europe Research and Innovation (Grant 101076915), and the EU 2DSPIN‐TECH Program (Graphene Flagship).

## Conflicts of Interest

The authors declare no conflicts of interest.

## Supporting information

Supplementary Material

## Data Availability

The data that support the findings of this study are available from the corresponding author upon reasonable request.
