# Supplementary material for: Gate‐Tunable Remnant Anomalous Hall Effect in Dual‐‍Proximity Graphene Heterostructures
Source: Small Sci. 2026 Sep 18;6(9):e70404. doi: 10.1002/smsc.70404 (PMC13588017; doi:10.1002/smsc.70404)
Supplement: Supplementary file 1 [file SMSC-6-e70404-s001.pdf]

Supporting Information

Gate-Tunable Remanent Anomalous Hall Effect in Dual-Proximity Graphene Heterostructures

Sayooj Satheesh, Alexandre Bernard, Thomas Naimer, Ernst Knöckl, Takashi Taniguchi, Kenji Watanabe, Jaroslav Fabian, Alexander Holleitner, Christoph Kastl*, and Marko Burghard*

S. Satheesh, M. Burghard
Max Planck Institute for Solid State Research, Heisenbergstrasse 1, 70569 Stuttgart, Germany

Thomas Naimer, Jaroslav Fabian
Institute for Theoretical Physics, University of Regensburg, 93040 Regensburg, Germany
Halle-Berlin-Regensburg Cluster of Excellence CCE, University of Regensburg, 93040 Regensburg, Germany

Takashi Taniguchi, Kenji Watanabe
National Institute for Materials Science, 1-1 Namiki, Tsukuba, 305-0044, Japan

Alexandre Bernard, Ernst Knöckl,Christoph Kastl, Alexander Holleitner
Walter Schottky Institute and Physics Department, Technical University of Munich, 85748 Garching 85748, Germany

Alexandre Bernard,Ernst Knöckl, Christoph Kastl, Alexander Holleitner
Munich Center For Quantum Science and Technology (MCQST), 80799 München, Germany
*: christoph.kastl@tum.de**,** m.burghard@fkf.mpg.de

Table of Contents

S1. Device parameters of all investigated trilayer and bilayer devices.
S2. R_{xy} vs. B dataset of $Cr_2Ge_2Te_6$/graphene control device for V_g=5-10 V.
S3. R_{xx} vs. B dataset of $Cr_2Ge_2Te_6$/graphene/WSe_2 trilayer1 (main text).
S4. R_{xy} vs. B dataset of $Cr_2Ge_2Te_6$/graphene/WSe_2 trilayer1 (main text).
S5. Densities and gate voltage-energy relation of device trilayer1 (main text).
S6. Characterization of the $Cr_2Ge_2Te_6$/graphene interfacial traps.
S7. Other device example: trilayer device 2 transport data.
S8. Anomalous Hall conductivity of model Hamiltonian within linear response theory.
S9. Landau fan diagrams of main device trilayer1.
S10. AFM data of main trilayer device 1.
S11. AFM data of bilayer device 2.
S12. Transport data on trilayer device 3

Table S1. Device parameters of all investigated trilayer and bilayer devices

**Sample**	**Layers (top to bottom)**	**Twist-angle (Gr-WSe_2)**	**Max. hysteresis (ΔR_{xy} at B = 0)**	**Density and mobility (n_0 and μ_0 at V_g = 0)**	**Other comments**
Bilayer1	70 nm Au<br>17 nm hBN<br>1L graphene<br>5 nm CGT<br>300 nm SiO_2	NA	32 Ω	$n_0 = 3.2 \times 10^{12}$ cm^{-2}<br>$\mu_0 = 2730$ cm^{2}V^{-1}s^{-1}	Fig. 2<br>Fig. S2, S6
Trilayer1	45 nm top hBN<br>4-5L graphite<br>35 nm hBN<br>1-3L WSe_2<br>1L graphene<br>8 nm CGT<br>300 nm SiO_2	22°	250 Ω	$n_0 = 3.2 \times 10^{12}$ cm^{-2}<br>$\mu_0 = 6000$ cm^{2}V^{-1}s^{-1}	Fig. 3, 4, 5<br>Fig. S3, S4, S5<br>Fig. S6, S8
Trilayer2	top hBN<br>6-7L graphite<br>hBN<br>1-3L WSe_2<br>1L graphene<br>few layer CGT<br>300 nm SiO_2	NA	120 Ω	$n_0 = 3.1 \times 10^{12}$ cm^{-2}<br>$\mu_0 = 6500$ cm^{2}V^{-1}s^{-1}	Fig. S7
Trilayer3	40 nm Au<br>hBN<br>1-3L WSe_2<br>1L graphene<br>few layer CGT<br>300 nm SiO_2	~2°	165Ω	$n_0 = 2.8 \times 10^{12}$ cm^{-2}<br>$\mu_0 = 7700$ cm^{2}V^{-1}s^{-1}	Fig. S12
Trilayer4	top hBN<br>graphite<br>hBN<br>2-3L WSe_2<br>1L graphene<br>few layer CGT<br>300 nm SiO_2	NA	260 Ω	$n_0 = 3 \times 10^{12}$ cm^{-2}<br>$\mu_0 = 12500$ cm^{2}V^{-1}s^{-1}	

S2. R_{xy} vs. B dataset of $Cr_2Ge_2Te_6$/graphene control device for V_g = 5-10 V

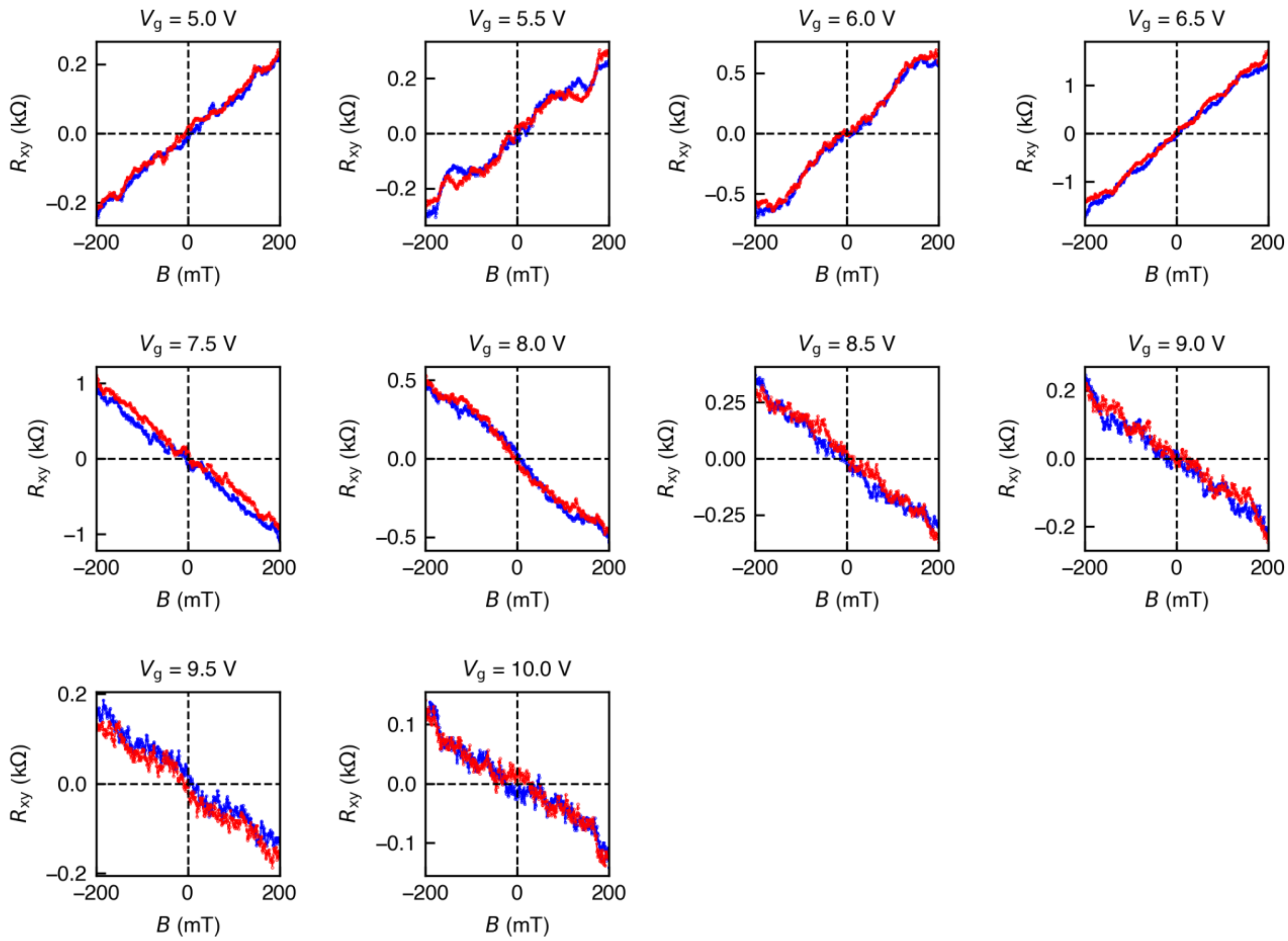

Fig. S2: Antisymmetrized R_{xy} vs. magnetic field at the indicated gate voltages between V_g = 5.0 V and V_g = 10.0 V (T = 4.2 K) for the control device bilayer1.

S3. R_{xx} vs. B dataset of $Cr_2Ge_2Te_6$/graphene/WSe_2 trilayer1 (main text)

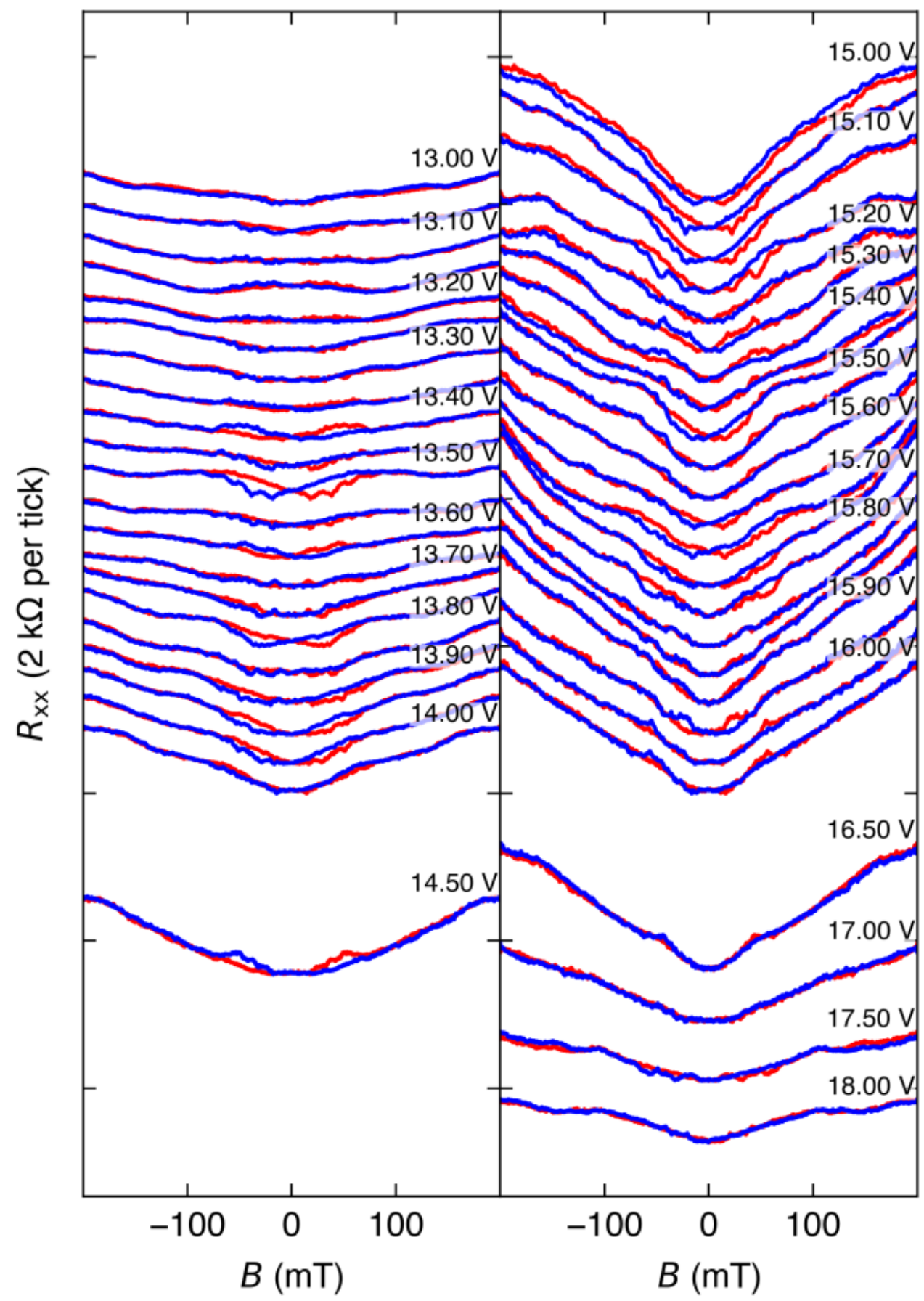

Fig. S3: Symmetrized R_{xx} vs. magnetic field at the indicated gate voltages between V_g = 13 V and V_g = 18 V (T = 4.2 K) for the main device trilayer1.

S4. R_{xy} vs. B dataset of $Cr_2Ge_2Te_6$/graphene/WSe_2 trilayer1 (main text)

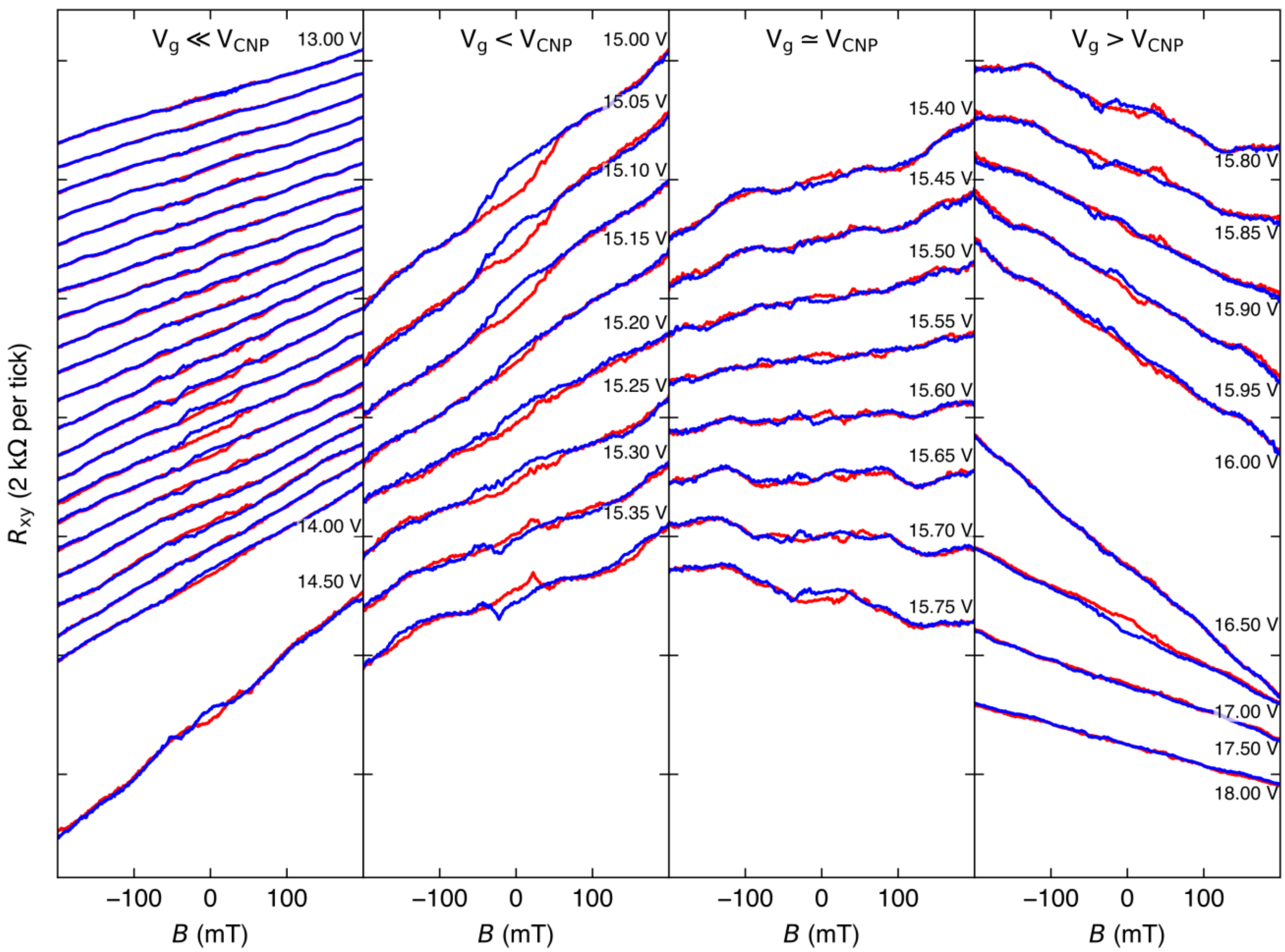

Fig. S4: Antisymmetrized R_{xy} vs. magnetic field at the indicated gate voltages between V_g = 13 V and V_g = 18 V (T = 4.2 K) for the main device trilayer1.

S5. Densities and gate voltage-energy relation of device trilayer1 (main text)

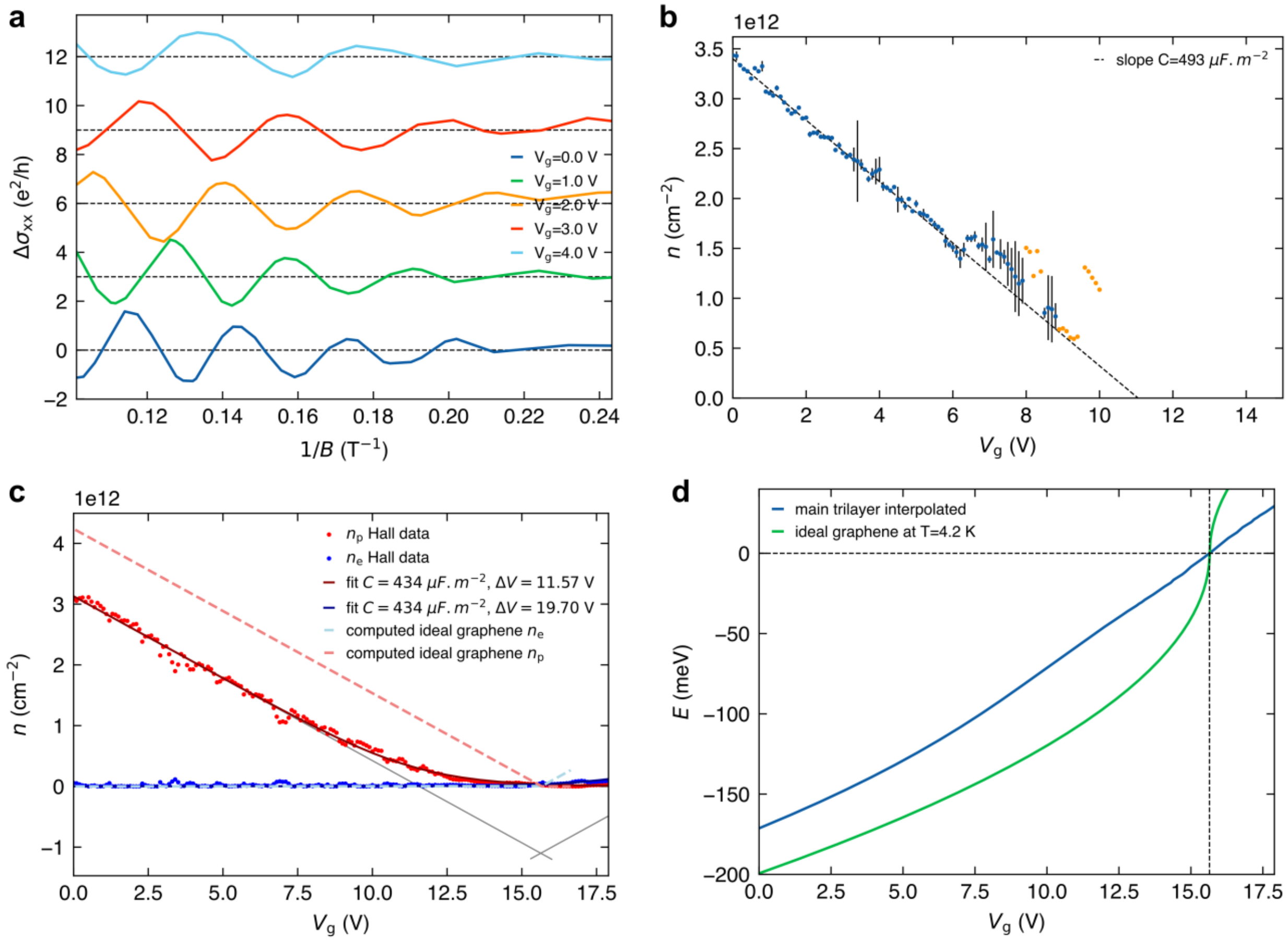

Fig. S5: Main device trilayer1. **a**. Shubnikov-de Haas oscillations for various V_g. Background was substracted. Curves are offset for visibility. **b**. Density extracted from SdH oscillations. Dashed line denotes a linear fit with resulting gate capacitance noted. **c**. Electron and hole densities from fits of both the longitudinal and transverse conductivities with the two-carrier model. Dark lines: fits with a phenomenological function. Grey lines: slope at high density assumed equal for electrons and holes yielding gate capacitance of C=434 μF.m^{-2}. The apparent contradiction with the ~35 nm hBN measured by AFM is solved when taking into account interfacial charge traps (see section S6). The electron and hole slopes do not intersect at n=0, which is a sign of interfacial charges. Dashed light lines: densities as a function of gate voltage from computed ideal graphene for T = 4.2 K, C = 434 μF.m^{-2} and $v_F = 8.3\text{x}10^5$ m.s^{-1}. **d**, Energy-gate voltage relation for ideal gating of graphene (green line) and from experimental densities (blue line). We use the relation shown by the blue curve anytime we plot experimental data versus energy. To obtain the blue curve, we use the fits of the experimental densities (dark lines in **c**) to get $n_e^{(\text{exp})}(V_g)$ and $n_p^{(\text{exp})}(V_g)$. We then compute $n_e^{(\text{th})}(E)$ and $n_p^{(\text{th})}(E)$ of ideal graphene, invert the relation $E\left(n_e^{(\text{th})}, n_p^{(\text{th})}\right)$, and compute $E(V_g) = E\left(n_e^{(\text{exp})}(V_g), n_p^{(\text{exp})}(V_g)\right)$.

S6. Characterization of the $Cr_2Ge_2Te_6$/graphene interfacial traps

When an insulating material is brought into proximity of a conducting material such as graphene, its near-interfacial traps (defects) can exchange carriers with the nearby conducting material. These traps act as donors or acceptors, and effectively screen the effect of the gate on the conducting material. The effect of these traps can be modeled by an effective capacitance C_{it} in series with the insulating layer capacitance C_{ins}.

We have:

$$C_{\mathrm{it}} = e\frac{dn_{\mathrm{it}}}{d\mu}$$

with $n_{\mathrm{it}}(\mu)$ the surface density of interfacial trap states and μ the electrochemical potential. In addition:

$$C_{\mathrm{it}} = eD_{\mathrm{it}}$$

with $D_{\mathrm{it}}(\mu)$ the density of interfacial trap states per unit of surface and energy. Assuming a constant density of trap states, the electrostatic charge neutrality condition between the gate, the traps and the graphene flake yields the following relation between the charge density imbalance in graphene $\left|n_{\mathrm{e}} - n_{\mathrm{p}}\right|$ and the gate voltage V_{g}[1]:

$$\left|n_{\mathrm{e}} - n_{\mathrm{p}}\right|\left(V_{\mathrm{g}}\right) = \frac{C_{\mathrm{ins}}}{e}\left(\left|V_{\mathrm{g}} - V_{\mathrm{CNP}}\right| + V_0\left(1 - \left(1 + 2\frac{\left|V_{\mathrm{g}} - V_{\mathrm{CNP}}\right|}{V_0}\right)^{1/2}\right)\right) \quad (1)$$

with V_{CNP} the charge neutrality point voltage of graphene and $V_0 = \left(1 + \frac{C_{\mathrm{it}}}{C_{\mathrm{ins}}}\right)^2 \frac{h^2 v_F^2}{8\pi e^3} C_{\mathrm{ins}}$.

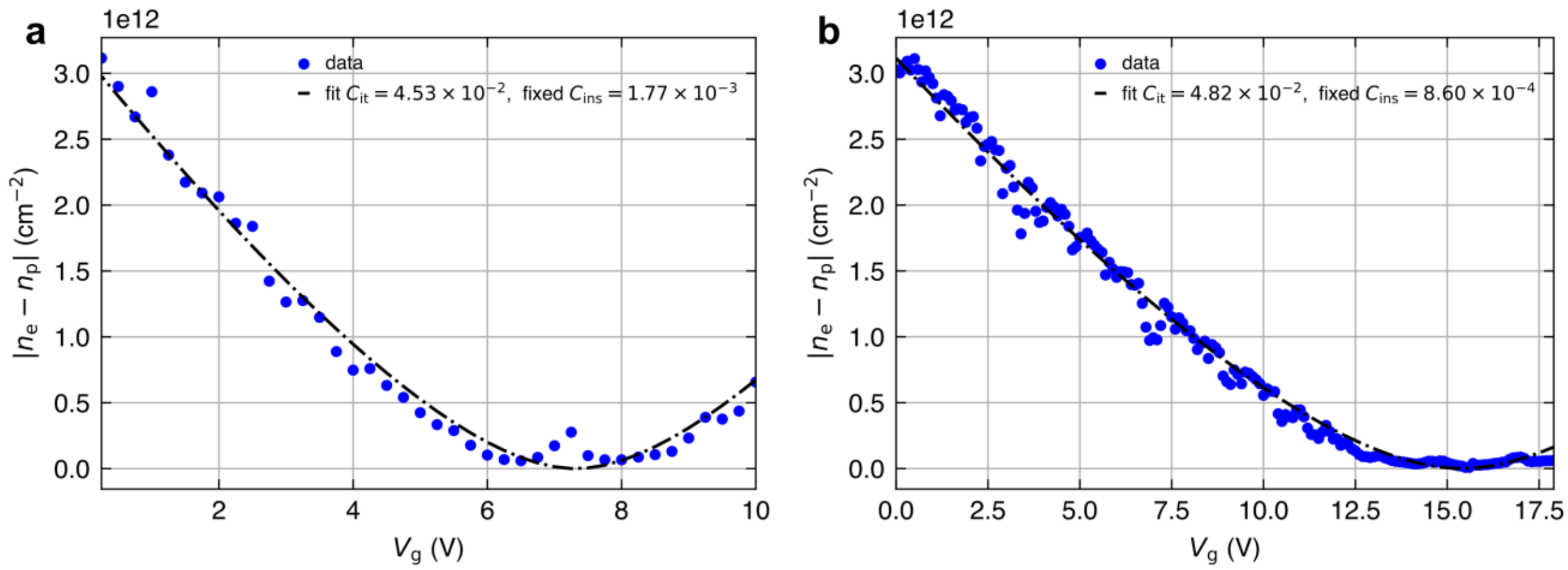

Fig. S6: Estimation of the interfacial traps capacitance (C_{it}) from density-gate voltage relations. $|n_e - n_p|$ is the charge density imbalance in the graphene layer, with n_e and n_p the electrons and holes densities, respectively. C_{ins} is the (standard) gate insulator capacitance estimated from the hBN thickness (measured by AFM) and assuming a relative dielectric constant $\varepsilon_{hBN} = 3.4$[2]. Blue dots: densities computed from fits of experimental conductivities at low B-field with the two-carrier model. **a**, Control device bilayer1. Black dashed-doted line: fit with formula (1), fixing $C_{ins} = 1.77$ mF.m^{-2} corresponding to $t_{hBN} = 17$ nm. The fitted $C_{it} = 45.3 \pm 1.2$ mF.m^{-2} corresponds to $D_{it} = (2.83 \pm 0.07) \times 10^{13}$ cm^{-2}.eV^{-1}. **b**, Main device trilayer1. Black dashed-doted line: fit with formula (1), fixing $C_{ins} = 860$ µF m^{-2} corresponding to $t_{hBN} = 35$ nm. The fitted $C_{it} = 48.2 \pm 0.2$ mF m^{-2} corresponds to $D_{it} = (3.01 \pm 0.01) \times 10^{13}$ cm^{-2} eV^{-1}. With this analysis, we find that CGT p-dopes graphene to $n \approx 3.2 \times 10^{12}$ cm^{-2} ($E_F \approx -170$ meV) and introduces interfacial charge traps with an energy density $D_{it} \approx 2.9 \times 10^{13}$ cm^{-2}.eV^{-1}.

S7. Other device example: trilayer device 2 transport data.

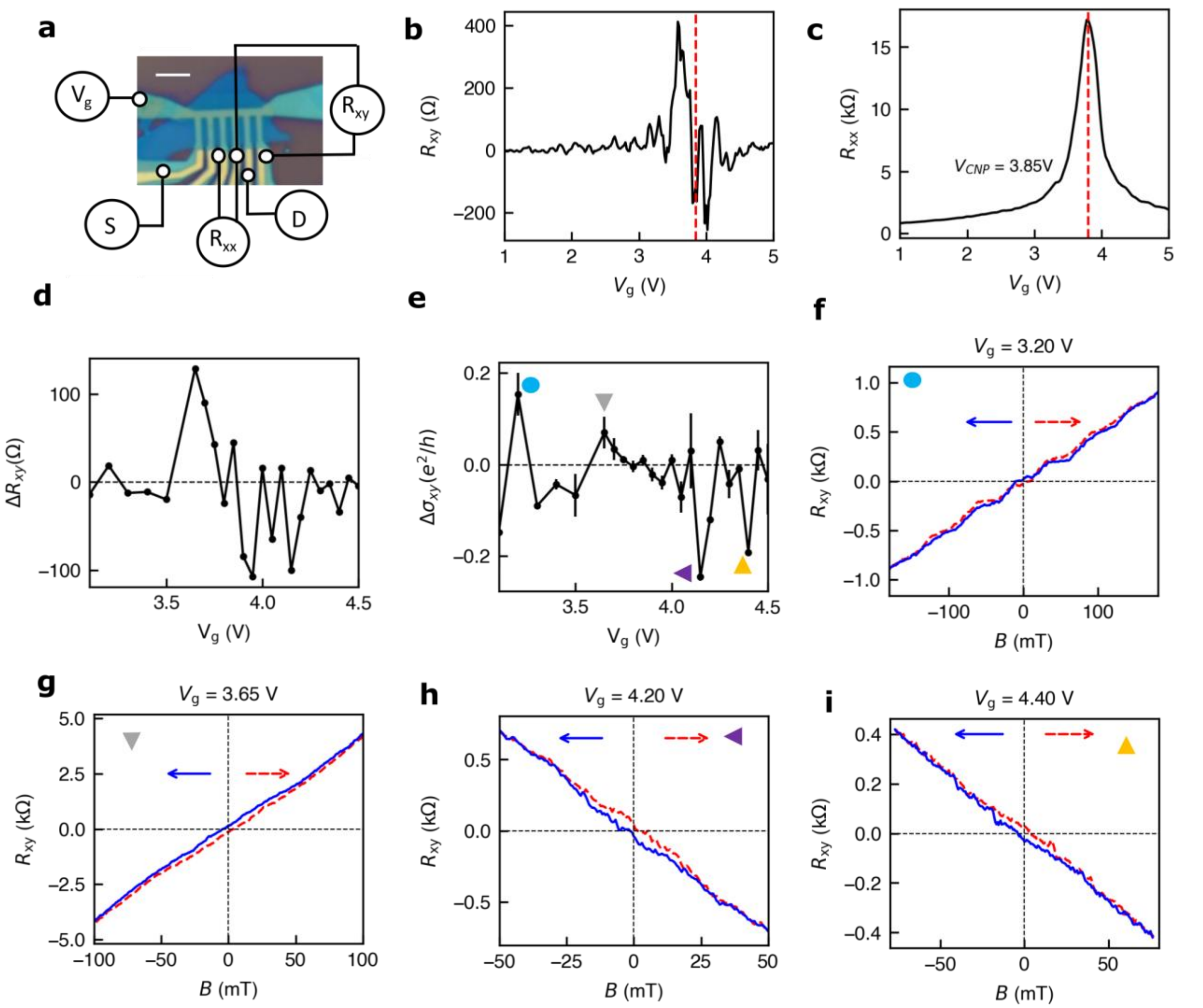

Fig. S7: **a**, Optical image of device trilayer 2 (scale bar: 5 µm). **b** and **c,** R_{xy} and R_{xx} vs gate voltage V_g measured via field training method. For positive field training measurement, B field is swept from 0 mT→100 mT→ 0 mT, and gate dependence is measured at 0 mT. For negative field training, B field is swept again from 0 mT→ -100 mT→ 0 mT and gate dependence is measured at 0 mT. The two measurements are then used to symmetrize the data. **d,** Gate-dependence of the R_{xy} hysteresis at B = 0 T. **e,** Gate-dependence of the anomalous Hall conductivity at B = 0 T. **f-i,** Antisymmetrized R_{xy} vs B-field at indicated gate voltages.The selected gate voltages correspond to the colored markers in panel **e** and span both sides of the charge-neutrality point. All the measurements were performed at T = 1.3 K.

S8. Anomalous Hall conductivity of model Hamiltonian within linear response theory

In order to describe the gating-dependent anomalous Hall conductivity of the proximitized graphene, we utilize the Kubo formula within the Smrcka Streda formulation on a Dirac model Hamiltonian.

We use an effective low-energy Hamiltonian to model the proximity effects of both CGT and WSe_2 on the graphene flake. The Hamiltonian $H(\boldsymbol{k})$ comprises the orbital part $H_{\mathrm{orb}}(\boldsymbol{k})$, the ferromagnetic exchange $H_{\mathrm{ex}}(\boldsymbol{k})$ and the spin-orbit part $H_{\mathrm{SOC}}(\boldsymbol{k})$. The latter is composed of the intrinsic spin-orbit coupling $H_{\mathrm{SOC,I}}(\boldsymbol{k})$ and the Rashba coupling $H_{\mathrm{SOC,R}}(\boldsymbol{k})$ [3,4]:

$$H(\boldsymbol{k}) = H_{\mathrm{orb}}(\boldsymbol{k}) + H_{\mathrm{ex}}(\boldsymbol{k}) + H_{\mathrm{SOC,I}}(\boldsymbol{k}) + H_{\mathrm{SOC,R}}(\boldsymbol{k}) \qquad (2)$$

$$H_{\mathrm{orb}}(\boldsymbol{k}) = \hbar v_F\left(\tau k_x \sigma_x - k_y \sigma_y\right) s_0 + \lambda_M \sigma_z s_0$$

$$H_{\mathrm{ex}}(\boldsymbol{k}) = -\lambda_E \sigma_0 s_z$$

$$H_{\mathrm{SOC,R}}(\boldsymbol{k}) = -\lambda_R\left(\tau \sigma_x s_y + \sigma_y s_x\right)$$

The orbital part describes the dispersion of the graphene Dirac cone linearized around the K/K'-point; accordingly, $\boldsymbol{k}$ is the electron wave vector measured from K/K'. It also includes a staggered potential (Dirac mass) λ_M, caused by the substrate's asymmetrical influence on the graphene A- and B-sublattices. v_{F} is the Fermi velocity of the Dirac electrons. σ_{i} and s_{i} (i=x,y,z) are the Pauli matrices operating on the sublattice (A/B) space and spin (↑/↓) space, respectively. σ_0 and s_0 are identity matrices. The valley parameter is $\tau = +1$ for K and $\tau = -1$ for K'. λ_{VZ}, λ_{KM} and λ_R are the valley-Zeeman, the Kane-Mele, and the Rashba spin-orbit coupling parameters, respectively. The Kane-Mele SOC is assumed to be irrelevant as it is usually limited to the intrinsic SOC of pristine graphene, which is on the μeV scale. For the AHE, the Rashba phase is irrelevant and therefore neglected in this approach.

We assume weak disorder scattering described by phenomenological parameter Γ. We calculate the anomalous Hall conductivity as

$$\sigma_{xy} = \frac{\hbar}{4\pi^2}\int d^2k \sum_{n\neq m}(f_{n,\boldsymbol{k}} - f_{m,\boldsymbol{k}})\frac{\mathrm{Im}\left(\langle n\boldsymbol{k}|-ev_y|m\boldsymbol{k}\rangle\langle m\boldsymbol{k}|-ev_x|n\boldsymbol{k}\rangle\right)}{(\varepsilon_{n\boldsymbol{k}} - \varepsilon_{m\boldsymbol{k}})^2 + \Gamma^2}$$

The integral is performed around K and K' points ensuring to cover all the relevant states (151x151 square grid with a side length of $\Delta k = 0.03\frac{eV}{\hbar v_F}$). $\varepsilon_{n\boldsymbol{k}}$ and $|n\boldsymbol{k}\rangle$ are the eigenenergy and eigenstate of band n as represented by the model Hamiltonian at $\boldsymbol{k}$. We set the broadening Γ of the states to $\Gamma = 0.1$ meV and the electronic temperature of the Fermi functions to 0.01 meV.

For the calculation in Fig. 5b), we used sensible model Hamiltonian parameters

$$\lambda_R = 3.0 \text{ meV}, \lambda_{VZ} = 2.0 \text{ meV}, \lambda_E = -3.6 \text{ meV}, \lambda_M = 0.9 \text{ meV}$$

and superimposed the differently weighted AHE signals of four cases with differently shifted Fermi levels (shifted by ΔE_F). Additionally, the sign of the ferromagnetic exchange parameters (and therefore the sign of the AHE signal) was varied for two of the cases. The total signal is:

$$\sigma_{xy} = \frac{1}{1+1.7+1.7+1}\left(-\sigma_{xy}(\Delta E_F = -14meV) - 1.7\cdot\sigma_{xy}(\Delta E_F = -9meV)\right.$$
$$\left.+1.7\cdot\sigma_{xy}(\Delta E_F = 9meV) + \sigma_{xy}(\Delta E_F = 14meV)\right)$$

For the blue curve, we additionally added a Gaussian broadening of 2meV.

S9. Landau fan diagrams of main device trilayer1

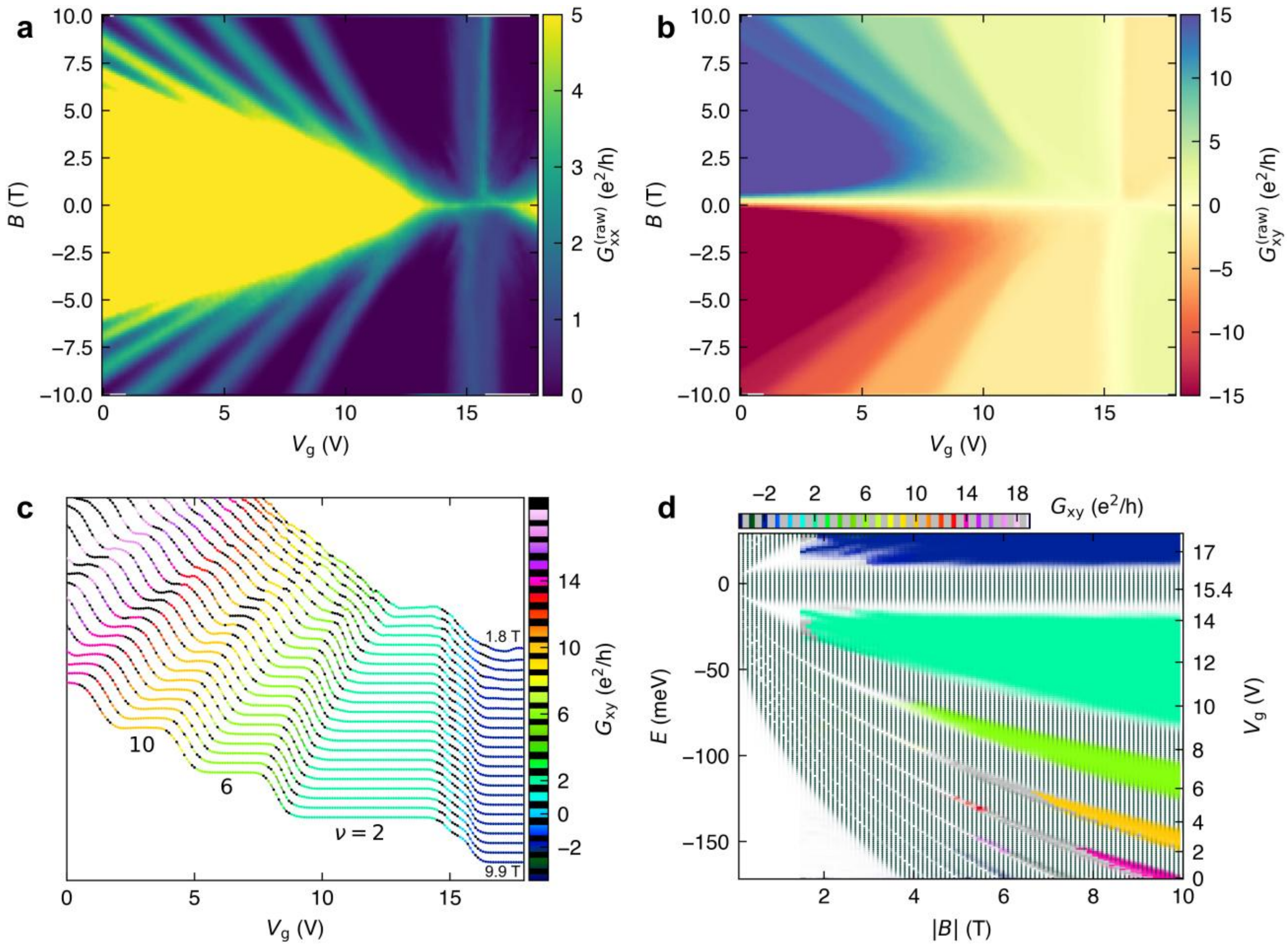

Fig. S9: **a** and **b**, main device trilayer1 longitudinal and transverse conductances $G_{\mathrm{xx}}^{(\mathrm{raw})} = R_{\mathrm{xx}}^{(\mathrm{raw})}/[\left(R_{\mathrm{xx}}^{(\mathrm{raw})}\right)^2 + \left(R_{\mathrm{xy}}^{(\mathrm{raw})}\right)^2]$ and $G_{\mathrm{xy}}^{(\mathrm{raw})} = R_{\mathrm{xy}}^{(\mathrm{raw})}/[\left(R_{\mathrm{xx}}^{(\mathrm{raw})}\right)^2 + \left(R_{\mathrm{xy}}^{(\mathrm{raw})}\right)^2]$ from unsymmetrized (raw) R_{xx} and R_{xy} data. **c**, Ridge-line plots of $G_{xy} = R_{xy} /[(R_{xx})^2 + (R_{xy})^2]$ as a function of V_g (T = 4.2 K), from symmetrized R_{xx} and antisymmetrized R_{xy}. Each curve corresponds to a fixed |B|, and is shifted with |B|. The colour scale highlights the expected conductance quantization at integer quantum Hall filling factors ν. **d**, Computed energies E of Landau levels for graphene with proximity-induced interactions λ_R = 3.0 meV, λ_{VZ} = 2.0 meV, λ_E = -3.6 meV, λ_M = 0.9 meV and $v_F = 8.3 \times 10^5$ ms^{-1}. The Landau levels (black dots) are phenomenologically broadened by a Gausssian with σ = 2.0 meV and filtered by a minimum slope *s*. The overlaid plateaus (coloured dots) are identified from **c** by their slope (with a maximum of *s*) and quantized values. The computed LL significantly deviate from the measured data for $|\lambda_R| > 12$ meV, $|\lambda_{VZ}| \notin [1, 4]$ meV and $\lambda_E \notin [-4.5, -2.5]$.

For the Landau fan analysis, we compute from the raw magnetotransport data the transverse conductivity G_{xy} via tensor inversion, using the symmetrized longitudinal and antisymmetrized Hall resistances R_{xx} and R_{xy}: σ_{xy} ($\equiv G_{xy}$) = R_{xy} / $[(R_{xx})^2 + (R_{xy})^2]$. Representing the fan in terms of

$G_{xy}(V_g,B)$ instead of R_{xy} facilitates the identification of QH structure, since integer states appear as extended, equally spaced plateaus at $G_{xy} = \nu e^2/h$, and the conversion reduces geometrical mixing and the classical low-field Hall background that can otherwise tilt features and obscure plateau boundaries.

We use an effective low-energy Hamiltonian to model the proximity effects of both CGT and WSe_2 on the graphene flake. We recall the Bloch Hamiltonian (2) for valley K ($\tau = +1$) and K' ($\tau = -1$) introduced in section S8:

$$H_\tau(\boldsymbol{k}) = \hbar v_F\left(\tau k_x\sigma_x - k_y\sigma_y\right)s_0 + \lambda_M\sigma_z s_0 + \lambda_R\left(-\tau\sigma_x s_y - \sigma_y s_x\right) + \tau\lambda_{KM}\sigma_z s_z + (\lambda_{VZ}\tau - \lambda_E + E_Z)\sigma_0 s_z$$

where we also introduced the Zeeman term $E_Z = g\mu_B B$.

In the $(A\uparrow, A\downarrow, B\uparrow, B\downarrow)$ basis:

$$H_\tau(k) = \begin{pmatrix} \lambda_M + \tau(\lambda_{KM}+\lambda_{VZ}) - \lambda_E + E_z & 0 & \hbar v_F(\tau k_x - ik_y) & (\tau+1)i\lambda_R \\ 0 & \lambda_M - \tau(\lambda_{KM}+\lambda_{VZ}) + \lambda_E - E_z & (1-\tau)i\lambda_R & \hbar v_F(\tau k_x - ik_y) \\ \hbar v_F(\tau k_x + ik_y) & -(1-\tau)i\lambda_R & -\lambda_M - \tau(\lambda_{KM}-\lambda_{VZ}) - \lambda_E + E_z & 0 \\ -(\tau+1)i\lambda_R & \hbar v_F(\tau k_x + ik_y) & 0 & -\lambda_M + \tau(\lambda_{KM}-\lambda_{VZ}) + \lambda_E - E_z \end{pmatrix}$$

Upon orbital coupling with the external magnetic field, quantized Landau levels form, and the Hamiltonian becomes [5,6]:

$$H_\tau^{B>0}(n) = \begin{pmatrix} \lambda_M + \tau(\lambda_{KM}+\lambda_{VZ}) - \lambda_E + E_z & 0 & -\tau\hbar\omega_B\sqrt{n} & (\tau+1)i\lambda_R \\ 0 & \lambda_M - \tau(\lambda_{KM}+\lambda_{VZ}) + \lambda_E - E_z & (1-\tau)i\lambda_R & -\tau\hbar\omega_B\sqrt{n+1} \\ -\tau\hbar\omega_B\sqrt{n} & -(1-\tau)i\lambda_R & -\lambda_M - \tau(\lambda_{KM}-\lambda_{VZ}) - \lambda_E + E_z & 0 \\ -(\tau+1)i\lambda_R & -\tau\hbar\omega_B\sqrt{n+1} & 0 & -\lambda_M + \tau(\lambda_{KM}-\lambda_{VZ}) + \lambda_E - E_z \end{pmatrix}$$

$$H_\tau^{B<0}(n) = \begin{pmatrix} \lambda_M + \tau(\lambda_{KM}+\lambda_{VZ}) - \lambda_E + E_z & 0 & \tau\hbar\omega_B\sqrt{n+1} & (\tau+1)i\lambda_R \\ 0 & \lambda_M - \tau(\lambda_{KM}+\lambda_{VZ}) + \lambda_E - E_z & (1-\tau)i\lambda_R & \tau\hbar\omega_B\sqrt{n} \\ \tau\hbar\omega_B\sqrt{n+1} & -(1-\tau)i\lambda_R & -\lambda_M - \tau(\lambda_{KM}-\lambda_{VZ}) - \lambda_E + E_z & 0 \\ -(\tau+1)i\lambda_R & \tau\hbar\omega_B\sqrt{n} & 0 & -\lambda_M + \tau(\lambda_{KM}-\lambda_{VZ}) + \lambda_E - E_z \end{pmatrix}$$

Where n labels the Landau levels and $\hbar\omega_B = \sqrt{2\hbar e|B|v_F^2}$ is the cyclotron energy for graphene.

S10. AFM data on trilayer device 1

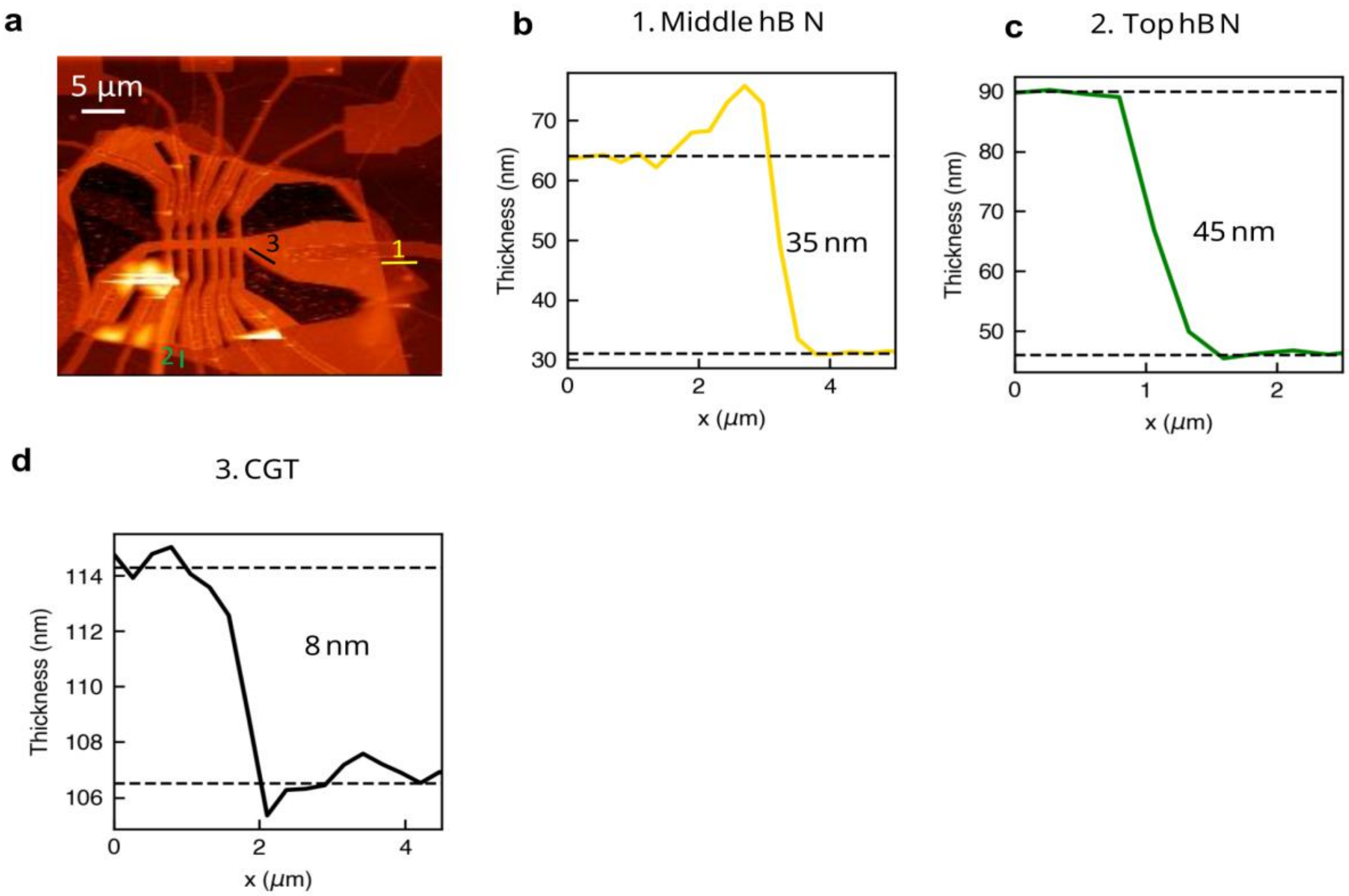

Fig. S10: **a**, AFM image of the trilayer device 1. **b-d**.AFM height profiles across the middle hBN, top hBN, and CGT flake.

S11. AFM data on bilayer device 1

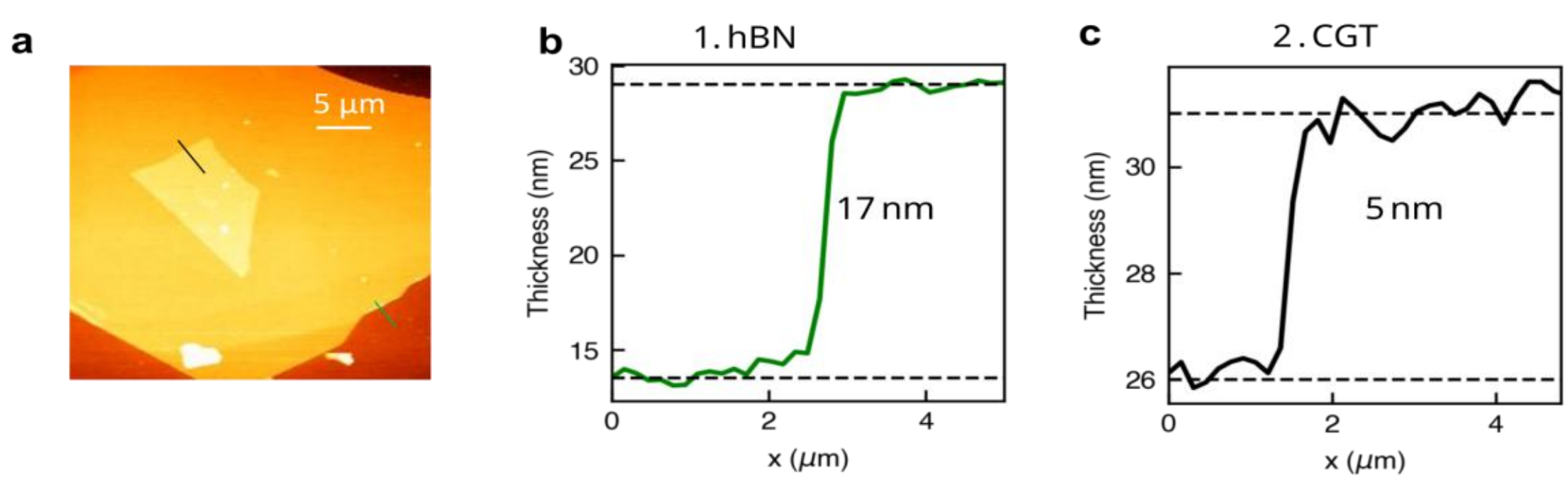

Fig. S11: **a**, AFM image of the bilayer device 1. **b-d**.AFM height profiles of top hBN, and CGT flake.

S12. Transport data on trilayer device 3

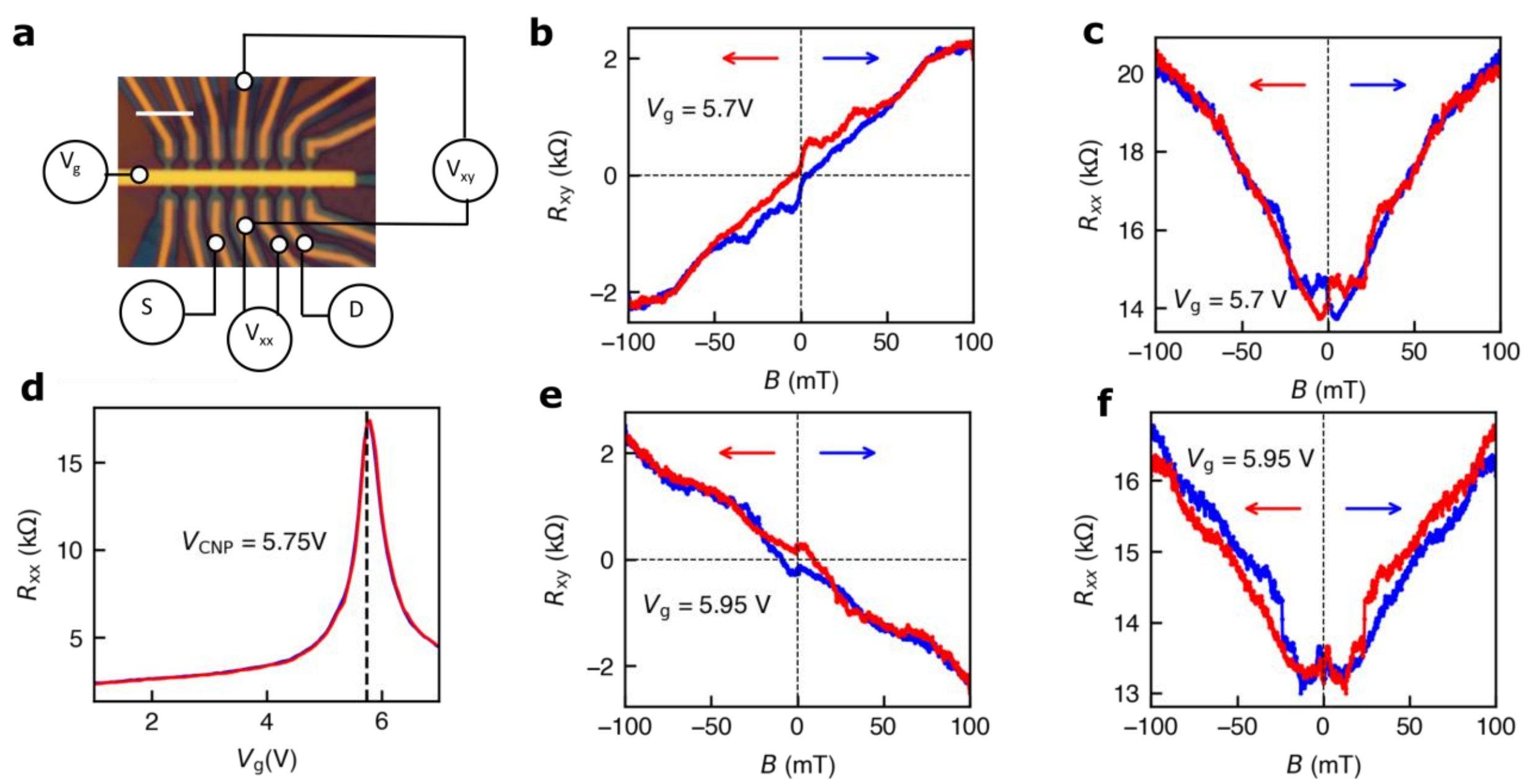

Fig. S12: **a**, Optical image of Trilayer Device 3 (scale bar: 5 µm). **b,e.** $R_{xy,}$ vs B, hysteresis measurement at gate voltage above and below dirac point. **d.**Transfer curve of the device. **c,f**. R_{xx} vs B, hysteresis gate voltage above and below dirac point. All the measurements were performed at T = 4.2 K

References

[1] Zebrev G. I., Physics and Applications of Graphene – Theory (2011) 475.

[2] Pierret, A., Mele, D., Graef, H., Palomo, J., Taniguchi, T., Watanabe, K., Li, Y., Toury, B., Journet, C., Steyer, P., Garnier, V., Loiseau, A., Berroir, J.-M., Bocquillon, E., Fève, G., Voisin, C., Baudin, E., Rosticher, M. & Plaçais, B. Dielectric permittivity, conductivity and breakdown field of hexagonal boron nitride. Mater. Res. Express 9, (2022) 065901.

[3] Zollner, K. & Fabian, J. Engineering Proximity Exchange by Twisting: Reversal of Ferromagnetic and Emergence of Antiferromagnetic Dirac Bands in $Cr_2Ge_2Te_6$. Phys. Rev. Lett. 128, 106401 (2022).

[4] Zollner, K., João, S. M., Nikolić, B. K. & Fabian, J. Twist- and gate-tunable proximity spin-orbit coupling, spin relaxation anisotropy, and charge-to-spin conversion in heterostructures of graphene and transition metal dichalcogenides. Phys. Rev. B (2023) 108, 235166.

[5] Frank, T. & Fabian, J. Landau levels in spin-orbit coupling proximitized graphene: Bulk states. Physical Review B (2020) 102, 165416.

[6] Rao, Q., Xue, H. & Ki, D.-K. Landau-Level Spectrum and the Effect of Spin–Orbit Coupling in Monolayer Graphene on Transition Metal Dichalcogenides. Phys. Status Solidi B (2024) 261, 2300397.
